# Caring for children with neurodevelopmental disability: Experiences from caretakers and health workers in rural eastern Uganda

**DOI:** 10.1371/journal.pone.0236488

**Published:** 2020-07-27

**Authors:** Gertrude Namazzi, Claudia Hanson, Christine Nalwadda, Moses Tetui, Margaret Nampijja, Peter Waiswa, James K. Tumwine, Helena Hildenwall

**Affiliations:** 1 Department of Health Policy, Planning and Management, School of Public Health, College of Health Sciences, Makerere University, Kampala, Uganda; 2 Department of Global Public Health, Karolinska Institutet, Stockholm, Sweden; 3 Department of Disease Control, London School of Hygiene and Tropical Medicine, London, England; 4 Department of Community Health and Behavioral Sciences, School of Public Health, College of Health Sciences, Makerere University, Kampala, Uganda; 5 Department of Epidemiology and Global Health, Umeå University, Umeå, Sweden; 6 Maternal and Child Wellbeing Unit, African Population and Health Research Center, Nairobi, Kenya; 7 Department of Pediatrics and Child Health, School of Medicine, College of Health Sciences, Makerere University, Kampala, Uganda; 8 Pediatric Emergency Department, Astrid Lindgren Children's Hospital, Karolinska University Hospital Huddinge, Stockholm, Sweden; University of Tampere, FINLAND

## Abstract

**Background:**

Long term outcomes of children with neurodevelopmental disability are influenced by the condition itself, available health services and caretakers’ coping ability to nurture the children which may be related to their beliefs and experiences. Most children with neurodevelopmental disabilities live in resource constrained settings. To inform design of contextually appropriate interventions, this study explored health workers’ and caretakers’ experiences in caring for infants with neurodevelopmental disability in rural eastern Uganda.

**Methods:**

A qualitative case study was carried out in December 2017 and involved in-depth interviews with 14 caretakers of infants with severe neurodevelopmental disability, and five health workers in Iganga/Mayuge Demographic Surveillance Site in eastern Uganda. The interviews with caretakers were conducted in *Lusoga*, the local language, and in English for the health workers, using a pre-determined open-ended interview guide. Data were analyzed using latent content analysis.

**Results:**

Caretakers described the experience of caring for children with neurodevelopmental disability as impoverishing and ‘imprisoning’ due to high care costs, inability to return to income generating activities and nursing challenges. The latter resulted from failure in body control and several aspects of nutrition and maintaining vital functions, coupled with limited support from the community and the health system. Many caretakers expressed beliefs in supernatural causes of neurodevelopmental disability though they reported about complications during and shortly after the birth of the affected child. Care-seeking was often challenging and impeded by costs and the feeling of lack of improvement. The health care system was also found to be incapable of adequately addressing the needs of such children due to lack of commodities, and human resource limitations.

**Conclusion:**

The caretakers expressed a feeling of emotional stress due to being left alone with a high nursing burden. Improvement in the health services including a holistic approach to care, improved community awareness and parental support could contribute to nursing of children with NDD.

## Introduction

The prevalence of neurodevelopmental disability (NDD) worldwide has not declined much for over two decades; estimated at 8.4% in 2016 compared to 8.9% in 1990 [1]. NDD comprise deficiencies in motor function, socio-emotional concerns, memory and learning problems, as well as deficits in sensory function during early child growth and development [2]. The commonest examples of NDD in children include attention-deficit hyperactivity disorder, autism, intellectual disabilities, cerebral palsy, learning disabilities, and impairments in vision and hearing.

Most of the disabilities are believed to be in low- and middle-income countries. However, access to information and professional care of children with NDD in especially low income countries (LICs) is still limited [3, 4]. Moreover, the long term outcomes of children with NDD may be influenced by the severity of the problem, available services and care, caretakers’ opinions on causes, as well as their attitudes and resilience in taking care of the children [5, 6]. In LICs, caretakers of infants with developmental delays and neurological deficits oftentimes have limited knowledge on the causes of the problem, how it can be managed and the likely future consequences, which may compromise care seeking [3, 7].

It is common in the African setting that there is a belief in spiritual cause of illnesses, such as for mental disorders, if the condition is perceived as complicated and not easily understood [8]. In Uganda, where 85% of the population are Christians, 12% Muslims, and 3% a mixture of other religions including traditional beliefs [9], consultations of traditional religious leaders for physical healing is prevalent. In a study conducted in south western Uganda, mental disabilities were believed to be due to intrinsic factors, punishment from God, or witchcraft, and those beliefs directed the treatment modalities [10]. However, care-seeking practices in LICs for children with NDD remain poorly understood. Earlier studies in Uganda and Malawi evaluated parents’ experiences about young children delivered in tertiary hospitals from urban setting [7, 11]. In Kenya, a study done in a rural setting, looked at care-givers’ experiences of school-going children [3]. The three studies report on caring burdens, emotional stress, financial difficulties, and challenges in availability of appropriate health services for children with developmental disabilities.

Health facilities in LICs have limited capacity to offer quality health services, particularly in rural areas [12, 13]. Where mental and support services exist they are usually managed by few providers, with inadequate supplies, and with insufficient information to caretakers on where to seek these services [11]. Health workers who run young child health clinics may lack knowledge and skills on how to care for children with NDD, or their focus is more on physical disabilities [11]. In addition, it is not clear what structures are in existence at community level to support families in care-seeking and nurturing of these children.

Additionally, previous studies report that caretakers of children with NDD may face social isolation and stigma from the community [7, 14, 15]. To cope, caretakers rely on social support to build self-efficacy in order to effectively care for their children [6, 16]. Understanding both caretakers’ and health providers’ perspectives in caring for children with NDDs can provide a foundation for development and implementation of programmes that empower families, communities and health facilities for timely and quality health service provision.

According to the Health Belief Model (HBM), timely care-seeking depends on one’s perception of severity of the condition, perceived barriers, perceived benefits, cues to action, and self-efficacy in care seeking [17]. HBM is a psychological model that was developed in the 1950s and has since undergone several adaptations, to explain utilization of preventive public health services based on individuals’ beliefs. HBM application is also important in designing health services with a holistic approach to care. This study therefore, with the aid of the HBM, examined the perceptions of the cause, care-seeking practices, and experiences, of caretakers with infants with developmental disability. In addition, it explored health workers’ perceptions of the management of these children, in order to contribute to the body of knowledge that can inform policies and design of contextually appropriate interventions for children with NDDs and their families.

## Materials and methods

### Study setting, design and participants

The study was approved by the Makerere University School of Health Sciences’ Institutional Review Board (SHSREC Ref: 2017–011) and the Uganda National Council of Science and Technology (Ref. SS4600).

This qualitative case study was conducted in December 2017 within Iganga/Mayuge Health Demographic Surveillance Site (HDSS) in Eastern Uganda. The HDSS site has 65 villages, with a total population of about 86,000 people served by one district hospital and 12 lower level health facilities. The district hospital also serves as a referral hospital for the surrounding five districts that lack hospitals, with a total population of over 1.5 million people.

The participants for this study were the caretakers of babies with NDD identified from a previous cross-sectional population-based study conducted by the same research group [18]. That study assessed 487 children aged 9–12 months to establish the prevalence and associated factors of NDD at population level in the HDSS. The study employed the Malawi Developmental Assessment Tool (MDAT) to examine four domains of development: Gross motor, Fine motor, Language, and Social behavior [19]. The child was considered as having NDD if he/she failed at least two or more of the parameters expected at his/her age in a given domain. We identified 12.7% (62/487) of infants aged 9–12 months, and resident in the HDSS with NDD, and 1.8% (9/487) were assessed as having severe NDD. Severe NDD was described as impairment in three or more developmental domains. The caretakers of these children with severe NDD; comprising nine mothers, and five fathers (who consented for interviews), were included in the current qualitative study. In addition, a convenience sample of five health providers from Iganga hospital were interviewed. The health workers were those found on duty from the pediatric ward, young child health clinic, and mental health clinics, which are service areas that handle vulnerable and disabled children, in the district hospital.

### Data collection procedures

Interviews with the caretakers were conducted by two research assistants, who were Ugandan university graduates with experience in qualitative data collection. The interviews with caretakers were conducted in *Lusoga*, the local language, using a pre-determined open-ended interview guide (Text box 1). The research assistants were trained in use of the in-depth interview tool by a social scientist and the first author. We pretested the tool on three caretakers of children with NDD identified through Village Health Teams (VHTs) from the nearby sub-county, outside the study area, to ensure there were no ambiguities in the questions asked. To reach the caretakers identified as guardians of a child with severe NDD, appointments were made through telephone calls. We audiotaped recordings of the interviews with the participants. Interviews were conducted at the caretakers’ homes, typically lasting 30–40 minutes. The first author supervised the research assistants during data collection and sat in all the interviews. GN did not require translation since she understands *Lusoga* which is quite similar to *Luganda*, her ethnic dialect.

**Text box 1. In-depth interview guide for caretakers with children having neurodevelopmental disability**

➢ Please tell us about your experience with pregnancy of (name): ANC attendance, any complications/danger signs and care received
➢ How was your experience of the birth of (name) and immediate postnatal period?
➢ What do you think is the problem with your child? *What do you think was the cause of the problem*?
➢ How did you know that your baby was having a problem?
➢ Please tell us about your experience with caring for your baby with this problem?
       *Probe for use of local herbs*, *traditional healers*, *health facility seeking*, *others*
       *Probe for facilitators*, *and challenges experienced in seeking the care*
       *Probe on whether the baby improved*
➢ How has your spouse supported you in care of this baby?
Probe for male involvement
➢ How do other people in the family/community behave towards your child because of his/her condition?
➢ Finally is there anything you want to be done to help you or other caregivers in taking care of babies with such a problem?

The interview tool explored perceptions of the causes of NDD, care-seeking practices, and caretakers’ experiences in taking care of these babies. Information was obtained from mothers about their experiences during pregnancy, labor and delivery, and during the postnatal period to understand circumstances surrounding the births of the children with NDD. Furthermore, we sought for information on community-level-perceptions of such children, their treatment and if any support was given to the caretakers and/or their children.

Interviews with health workers were conducted by the first author in English. The aim of these interviews was to assess the health providers’ opinions on how caretakers look after children with NDD, any challenges faced in care provision, and to understand the available health services for children with NDD.

### Data management and analysis

The interviews conducted in *Lusoga* were directly transcribed into English by the research assistant who conducted the interviews, a *Lusoga* speaking

**Fig 1:**
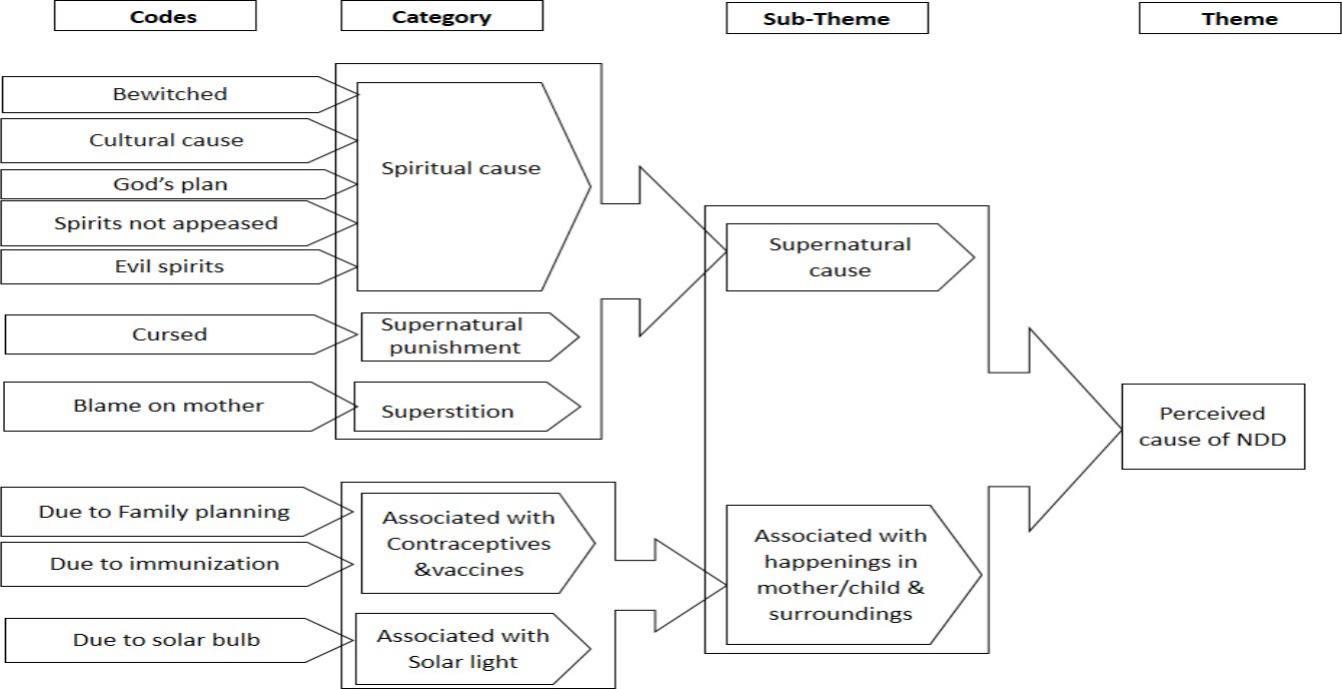
An example of qualitative analysis process for the theme perception of causes of neurodevelopmental disability.

Ugandan scientist, while health worker interviews were transcribed by the first author. Once the transcripts were ready for analysis, they were exported to Atlas Ti version 8. Data were analyzed using latent content analysis methods in order to yield a deeper understanding of participants’ experiences and opinions [20]. The transcripts were coded separately by three authors (GN, MT, and CN) who independently came up with labels to attach to portions that appeared to indicate important caretakers’ perspectives. Analysis started with **reading and re-reading of the transcripts in full, to gain** familiarity and to **identify connotation units. This involved** an iterative process that finally yielded meaningful units, which were then coded. Care was taken to ensure that the codes were as close as possible to the meaningful units identified. The codes were then discussed through review meetings held after which the trio reached consensus. MT is a Ugandan social scientist and CN has experience in qualitative studies on child health from the study area. After sharing the codes with other authors, a process of grouping the codes followed. GN led the process with guidance from HH, by combining codes into groups individually, and then discussed with MT and CN until consensus on fullness of the groups was reached. Linkages within the groups were identified, leading to categories. The categories were later grouped based on the predefined overarching themes of the study, including perceptions of the cause of the NDD, care-seeking practices for children with NDDs, the health system challenges, and the experiences of caretakers in caring for children with NDDs. Interpretation of the findings was inspired in part by the HBM model which enabled us to assess the findings in light of behavioral change triggers. Fig 1 illustrates the movement from codes to themes for the theme perception of causes of NDD.

### Informed consent statement

Before conducting the interviews, informed written consent was obtained from all the participants. Caretakers who could not read or write consented with a witnessed thumb print. The caretakers were encouraged to seek care for the children at the regional referral hospital where a multidisciplinary approach to care (physiotherapy, nutrition, counselling, and treatment of repeated illnesses) was possible. They were given shillings 10,000/ = (US$3) to aid in transportation of the children to and from the hospital.

## Findings

**Fig 2:**
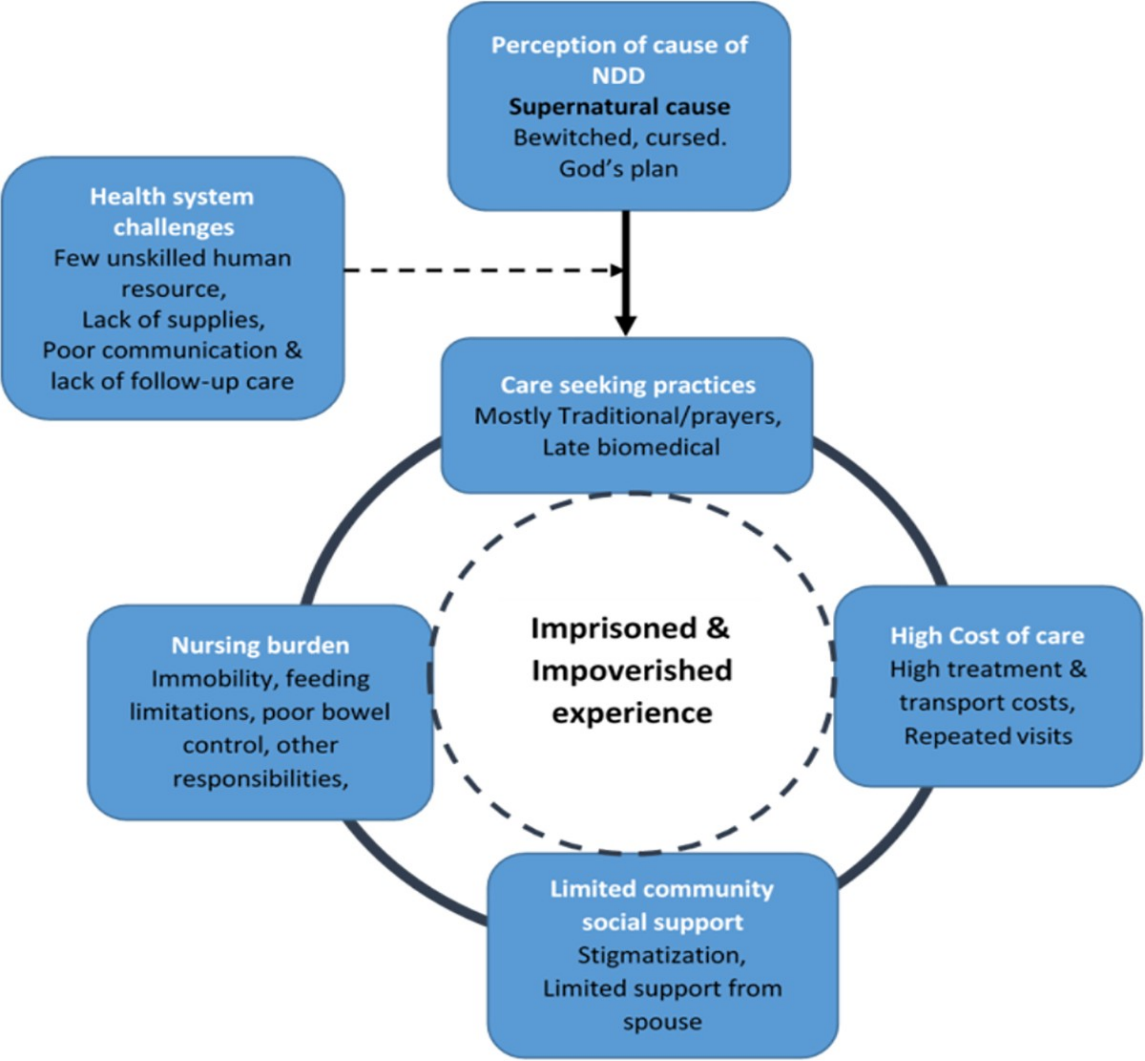
Caretakers’ perceptions and lived experiences of caring for children with neurodevelopmental disability.

Our overall results revealed an interconnected relationship of the four broad themes: 1) the imprisoning and impoverishing experience of caretakers with children having NDD, 2) Caretakers’ perception of the cause of the NDD as supernatural, 3) the care-seeking practices which were mostly from traditional healers, and 4) the weak health system that was unable to provide quality care that addresses caretakers’ needs and those of their children with NDD (Fig 2).

### Imprisoning and impoverishing experience of caretakers

Caretakers’ experience was broadly expressed as impoverishing and ‘imprisoning’ due to overwhelming nursing burden, financial constraints, limited community support, and stigmatization.

The child’s body weakness and immobility, such as failure to sit, crawl or walk, was one of the major challenges expressed by the caretakers. Caretakers reported having to carry the children most of the time on their own, which compromised their ability to engage in income generating activities, rest, and offer adequate nursing care.

“..*at times I prepare gardens for people to get some money*……*so I don’t even dig anymore because he doesn’t sit*, *stand or walk*, *and doesn’t want to be carried on the back*…… . . *before getting this child*, *I could hire land and farm on it but now I can’t*.*” Mother 3*

In addition, the caretakers reported challenges in feeding the children and toilet habits, as well as repeated illnesses such as respiratory infections. Some mothers described the situation as ‘imprisonment’.

“*I have gone through a very hard time*, *call it prison*! *Because now the baby is a year but is just starting to sit*, *cannot drink from a cup*, *and he gets ill frequently*. *He doesn’t talk*, *he doesn’t cry or do anything*. *…*..*he passes stool in that very place he is in*.*”* Mother 4

Nursing children with NDD was reported as one of the most challenging experience to the extent that one male caretaker even wished he could sell off his own child.

Caregivers expressed challenges with the finances required for treatment and care of these children. They mentioned high costs of treatment, transport to seek for care and expensive food for their children.

“*As I said*, *what these babies use for treatment is expensive*, *and the transport fees (to hospital) are high*, *and you may look for money but you don’t get it so if there is an organization to help me…*.*”* Mother 4

In addition, there were repeated visits to the hospital. This, coupled with failure to return to work in order to nurse their children was considered as impoverishing, as the expenses did not reduce over time.

“*I reached a time when I was left with nothing*, *take this*, *take that and all that time when we were in hospital I wasn’t working…*.*and this child didn’t feed very well so we had to buy milk and feed him on milk only which was costly*.*”* Father 2

#### Social and financial support

Most mothers reported lack of social and financial support from their spouses. Two mothers reported being supported by their parents, and these were young mothers. Older mothers obtained funds through working for others or getting loans from saving groups. However, looking for work to get money was reported as challenging given the fact that, often, mothers had nobody to leave their sick children with, coupled with other home responsibilities.

“*I have so many hardships with getting help because even if I ask for help no one helps me*. *That baby doesn’t breast feed any more but my husband cannot even buy a cup of milk*. *I get money from SACCOS to take care of him since I no longer work*. *The baby doesn’t sit down*, *he needs to be carried all the time yet the other children want to eat*.*” Mother 3*

On the other hand, all men who were interviewed reported to be very supportive of their wives in taking care of the sick children.

“*I do everything necessary*, *at home I treat her and carry her*. *I feed her and anything she needs I buy*. *I also play with her if I want”* Father 1

Also, two women described their spouses as very supportive. Two men reportedly lost their sources of income as a consequence of their children’s disability.

“*…He loves him but because he became poor (due to closure of his business) because of him (child) when he got sick*, *he doesn’t want to help*. *I have not received any support from my husband since his business closed*.*”* Mother 5

Reports from health workers were to the contrary; they revealed that only mothers and grandmothers bring children for care. They believed that many times the male counterparts were not supportive and could even abandon the children whom they considered a curse to the family. Health workers mentioned that, sometimes, even the mothers abandon the babies with NDD in order to get married elsewhere or in pursuit of income.

“*…recently a grandmother brought a three year old boy here in the hospital*. *His main problems were fits and when not fitting he would drum excessively*. *We asked the grandmother on the whereabouts of the mother of the child and she mentioned that the mother abandoned the child at one year and got married elsewhere*.*”* Health worker 3

#### Perceived community attitudes

The study findings revealed mixed perceptions of community attitudes and behavior towards the children with NDD and their parents. Some community members were reported to be sympathetic and advised on what the children were suffering from, and where to seek care. Yet, other mothers felt so stigmatized that they could not participate in social functions. This stemmed from either being blamed for having given birth to a disabled child or the shame the community members attach to having a child with NDD.

“*The way people can laugh at you*! *That you delivered a ‘mawungu’ (mentally retarded)*! *I stopped going for ceremonies*. *I would rather stay at home and send my contribution than face people staring and laughing at me*. *They say that I brought a curse to this family*.*”* Mother 4

However, one male participant said that such community attitudes and behavior are widespread at the beginning of the illness but later people stop interfering or caring about other peoples’ disabled children.

“*Once a person is sick for a long time people stop being concerned*, *but at first when something has just happened*, *they can behave anyhow*.*”* Father 5

### Caretakers’ perceptions of the cause of neurodevelopmental disability

Most (13/14) caretakers perceived their child’s NDD to be due to supernatural causes, such as witchcraft, curses or God’s plan. Caretakers reported that the children had been bewitched by people whom they were having poor relations with. Others thought that the NDD was due to curses from senior members of the family including in-laws and co-wives, or failure to appease the spirits.

“*This baby has two main problems*: *the first one*, *the paternal grandmother of this baby did not like me and because of that she was not happy for my baby to be given her name*, *and that is how the baby’s problems started*. *The grandmother was supposed to prepare a meal to remove the curse but she did not and that is why the child is not getting any better*. *The second problem*, *my co-wives did not feel good about my marriage and they turned all their anger against this child and made him fail to grow well*.*”* Mother 1

The caretakers reported on biomedical health problems, either at the time of birth, or in the immediate postpartum period, but they did not link the NDD to these complications. For instance, three babies reportedly had difficulties in breathing and failed to cry at birth following prolonged or difficult deliveries. In response, caretakers reported to have poured cold water on the neonates in a bid to stimulate the cry.

"..*for this one (pregnancy) I took three days in labour*, *but I was given herbs that helped me to deliver*. *The baby failed to cry*, *we poured cold water to make her cry; she was able to make some sounds though she rarely cries*.*”* Mother 3

Two mothers reported that the infants had developed fever and convulsions in the first week after birth, which necessitated admission for over two weeks, and other complications setting in later. While, one of them mentioned the cause of NDD to be due to the fever and convulsions, she left the hospital when the child still had convulsions and later took the child to a traditional healer.

The findings showed that, in some situations, whatever care the parents had tried, the baby was not showing any significant improvement. This caused the parents to resign and relate the cause of disability to God’s plan.

“*…I have treated him*, *but he doesn’t get fine so I get fed up…*..*so I decided to just care for him at home*, *maybe it was God’s plan*. *… I don’t know because I think God had his plan like that*.*”* Father 1

Disabilities were also reported to be associated with what was happening to the caretakers, to the children, or to their surroundings. In two cases, caretakers perceived the use of contraceptives or immunization to be responsible for NDD. While in another case, the parents associated the child’s condition to the use of solar light in their house.

“……… .*I thought that the way her eyes look*, *were due to a solar bulb in the house; because once I switch on I don’t switch off for some time so my mother told me that the solar bulb affected the child’s eyes*…*”* Father 2

### Care-seeking practices for children with NDD

Caretakers reportedly sought various treatment modalities to ensure the wellbeing of their children. Most caretakers used herbs, traditional medicine, and sought care from witchdoctors as the first plan of action due to the perception that the cause of the child’s condition was supernatural.

During one of the interviews, we noticed a key tied on a string around the waist of the child and asked the mother for the rationale, to which she responded that a key or safety pin tied around the waist prevents witchcraft and evil spirits from reaching the child.

Some caretakers resorted to prayer, since after trying other methods there was no improvement recorded. However, the caretakers reported challenges of travelling long distances to reach the churches and fasting for long periods of time.

“*Only prayers worked because they (health workers) told us there was no illness*. *So after some time of praying he started improving*. *So we observed some change and we continued praying and at one year he has started sitting and we had hopes that he would improve but where we pray from is very far*.*”* Mother 9

One mother who reported using biomedical care for her child with NDD complained of high transport costs due to frequent visits and high costs of medicines.

“*Okay I have ever gone to Jinja*, *a British founded hospital and he was treated once but because of transport costs I didn’t go back*. *They used to make exercises which helped*, *in that he started sitting*. *…but I failed to go back there because of high transport costs*.*”* Mother 6

Five caretakers used biomedical interventions for subsequent childhood illnesses, such as respiratory infections. Some of the caretakers reported buying syrups from drug shops for such illnesses, and did not visit the health facility. On the other hand, one caretaker reported taking the child to the witch doctor, when the child had difficulty in swallowing, in order to remove what he considered was in the throat stopping the child from swallowing.

“*You see they (evil people) sent him ‘ebiwala’ (foreign bodies sent through supernatural means)*. *Yes*, *because he wasn’t eating and he had been vomiting anything he eats but when they (ebiwla) were removed there was some improvement with the vomiting*.*”* Mother 6

The interviews also revealed some unconventional methods of managing children with NDD in this setting. For instance, when one child was unable to control the neck the parents were advised to dig up a hole and place the baby in there. Also, due to body weakness and immobility, such as inability to sit, the caretakers reported to have forcefully made the child to sit up.

“*At three months old*, *the baby became weak and even the neck was just bent and the eyes were not looking good*. *He has spent a full year without sitting but we decided to force him to sit and the bones became strong*.*”* Father 3

Reports from health workers revealed that few caretakers of children with NDDs come to the health facility to seek care, and if they do, they do so late after birth. Health workers mentioned that the children with NDD may be brought to the health facility for the first time at around two years of age after failing to improve. In addition, health workers confirmed that caretakers of children with NDD often sought care from traditional healers due to widespread beliefs in witchcraft.

### The weak health system

The data revealed challenges in the health system while providing care to children with NDD. This was mainly due to the few and unskilled health providers, lack of supplies, limitations in communication and lack of follow up of high risk children delivered from health facilities.

The health workers interviewed reported limited knowledge regarding the management of NDD. None of the nurses had received any training in that area, neither during the pre-service training nor as on-job training. They mostly treated recurrent illnesses, and sent children with physical disability for physiotherapy.

“*We have not received any training about neural development*, *not even in the nursing schools*. *What we know and do is to ensure mothers take folic acid during pregnancy for proper growth of the baby*. *Those with complications we tell them to come back for review but many do not turn up*. *If the child comes back with any abnormality like cerebral palsy we send them for physiotherapy*.*”* Health worker1

Health workers also reported inadequate human resource and supplies, and a lack of essential equipment and appliances in the physiotherapy department as well as in the outpatient mental health clinic. The outpatient mental health clinic had two nurses, one per duty shift and provided care for all age groups. Similarly, the physiotherapy department was understaffed, with only one physiotherapist.

“*There is one person working in the physiotherapy department*, *we know patients are there in the community but how to get them here is a big problem*. *A few cases of cerebral palsy come*. *They come when they are between two and three years*. *The parents first try traditional healers*, *when they do not see improvement then they come to hospital*.*”* Health worker2

#### Poor communication and lack of follow up by health workers

Two caretakers reported the need for appropriate information and care from the health system, which was not received. In addition, the caretakers’ reports were in agreement with those of the health workers on the fact that none of the children were ever followed up at community level, to track the outcome, have early identification of the NDD, or counselling. The health workers cited lack of human resource for failure to do community follow up.

Three mothers reported having been visited by the VHT, however, the VHTs were not helpful in giving advice to the mothers, and lacked the right information to help caretakers on how and where to manage the children from. Consequently, mothers turn to community members for help in identifying the problem and advice on where to seek care from.

“*A VHT came with a book and I explained to her the condition of my baby that it was not good*………*so she told me that I will ask and I advise you where to go but she did not come back*. *I suffered with this baby so much because his neck couldn’t support the head*.*”* Mother 8

## Discussion

This study has revealed the challenging experience of caring for a child with NDD, described as impoverishing and imprisonment, due to the high nursing burden, emotional stress and limited support from the community and the health system. In addition, there is a strong belief among caretakers of supernatural causes of NDD, which led them to seek care mostly from traditional healers and religious leaders.

The caretakers described the experience of caring for children with NDD as ‘imprisonment’ due to the high nursing care burden of those children resulting from poor body control and maintenance of vital functions. These findings are similar to those of a study in Malawi where caretakers depicted the scenario as ‘grounded’ [11]. In addition, caretakers were unable to engage in income-generating activities, yet the care of such children required a lot more funds resulting in impoverishment of already poor families, hence causing a lot of anxiety and stress, commonly reported among parents with chronically ill children [21–25]. The nursing challenges coupled with minimal or no improvement in children’s condition left the caretakers with a sense of hopelessness and desperation which might cause abuse, neglect and deprivation of those children [26].

Using the HBM framework, the challenges of nursing the children and the increased transport and treatment costs were some of the barriers to care-seeking that caretakers with children with NDD faced. Moreover, failure to return to income generating activities, and the poor economic status of rural families made it difficult for them to sustain treatment. In the study setting, the primary caretakers of sick children are usually women. However, the multiple roles and responsibilities of women in caring for their families with a child with NDD, and limited male involvement in care, left the women overwhelmed [3, 7]. Thus, there is a need to better support caretakers as they face challenges with repeated illnesses, nutrition, and several body functions of children with NDD.

The perceived benefits of health services provided also determine the clients’ care seeking practices [17]. In this study the caretakers’ perceived benefits were low due to a weak health system that was, largely, unresponsive to the needs of the caretakers and their children with NDD. The health workers confirmed the limited quality of health services at the facilities due to inadequacies in health providers’ skills, low staffing levels, and unavailability of essential commodities. In addition, there was lack of mechanisms to follow up at-risk babies at community level for early identification of complications [27]. According to the Ministry of Health guidelines in Uganda, the VHTs are expected to carry out this role [28]. Yet, reports from caretakers revealed that the VHTs who visited them lacked relevant information. This left some caretakers helpless and they consequently sought help from traditional healers, herbalists and religious leaders. Such health system weaknesses exacerbate the innate effect of traditional and cultural beliefs in caring for children with NDD. On the other hand, some caretakers still approached the health facilities for care of their children with NDD. This is in agreement with the notion that extreme situations lead to care seeking [29]. There is, therefore, a need to strengthen the health system in provision of quality rehabilitation services while considering the potential barriers experienced by caretakers, at the same time respecting their beliefs and values [4].

In this study, although caretakers reported on complications during birth and the postnatal period, most of them did not relate those with the child’s NDD, but instead referred to supernatural causes for the child’s condition. This could be explained by the widespread beliefs in supernatural powers as causes of mental disorders held over decades in the country [10]. Previous evidence also suggests that seeking care from biomedical services was influenced by traditional and cultural beliefs [30, 31]. This could also be a result of ineffective communication from the health workers to the parents of those children at the time of discharge from the health facility. Parents have a right to information of the expected prognosis of the children following a difficult delivery or any perinatal or postnatal insult. In the absence of the correct information, caretakers sought support from churches and traditional healers. These findings are in agreement with the African Belief model which describes mental illnesses as challenging to Africans, and hence attributed to spiritual causes [8]. However, to a few caretakers spirituality was one of the coping strategies in caring for children with NDD which is consistent with the family resilience framework [32].

On the other hand, our findings differ from a previous study conducted in urban Uganda [7] where mothers showed a good understanding of biomedical causes of NDDs. This may be due to differences in level of education of caretakers, and may also reflect equity issues in access to information and care between the urban and rural population. It is therefore, important that programs aimed at creating awareness to address information needs regarding NDD are set up nationally. Such programs can be integrated into existing maternal, newborn and child health services such as counseling during antenatal visits, at discharge from health facilities and during immunization sessions. In addition, community-wide awareness is important to address the social isolation and stigmatization, improve on social support and hence increase on the resilience of caregivers in taking care of children with NDD [6]. Nevertheless, prevention is better than rehabilitation [33] and most disabilities in LICs can be prevented if timely quality health services are provided [34]. Thus, affordable quality obstetric and neonatal services should be assured for all and at all levels, including rural settings, to further minimize preventable causes of NDD nationwide.

### Methodological considerations

This study focused on caretakers of young children with severe NDD. Perceptions, and experiences among caretakers with children with moderate or minor NDD may be different. In addition, caretakers’ experiences with older children having NDD would vary considering that those children are supposed to start school. Furthermore, the sample of children assessed with severe NDD was small, limiting the number of caretakers interviewed in this study, and may have had limitations on data saturation. However, we applied a qualitative case study methodology which utilizes small numbers of study participants to give a detailed description of the phenomena in a given context. This study was done in a rural area; given that over 80% of the population in Uganda is in the rural setting makes this study relevant and very important in understanding the parents’ experiences, perceived causes, and care-seeking practices for children with NDD in Uganda.

Further methodological consideration in interpretation of the study findings should be based on the fact that GN, who led the process of data collection and analysis is a medical doctor with a masters’ degree in Public Health. By the time of this study, she had 10 years of experience in maternal, newborn, and child health research at community and health facility level in Eastern Uganda where this study was conducted. Therefore, she had an adequate understanding of the context of the study setting. We acknowledge the limitation of the author medical team which might have led to a stronger recognition of the health system factors, however, a co-author who is a social scientist was involved in analysis and interpretation of the study findings. We aimed to ensure trustworthiness through a reflexive approach to analysis process, and provision of the study participants’ quotes for accurate illustration of their views and opinions.

## Conclusions

Caretakers experienced physical, financial, emotional, and nursing barriers to care of their children with NDD and received limited support from the community and the health system. This led to a feeling of being impoverished and ‘imprisoned’, causing emotional stress with associated risk of neglect of the children. Caretakers of infants with NDD perceived neurodevelopmental disability to be due to supernatural causes and therefore sought care from traditional and spiritual healers. However, some caretakers expressed the need for information and sought care for their children from the health system.

Our findings point to the need to improve the health system’s readiness to care for children with NDDs in rural settings in LICs with a holistic approach. Counselling, psychosocial support, stimulation, and rehabilitation services can be integrated into existing maternal, newborn and child health services to ensure constant availability and accessibility of such critical services. Community based programs for raising awareness are also needed to address the information needs on the causes of NDD while respecting the caretakers’ beliefs and values, in order to improve social support and resilience of the caretakers in coping with adversity of NDD.

## Supporting information

S1 Data (XLSX)
