# Correction: Caring for children with neurodevelopmental disability: Experiences from caretakers and health workers in rural eastern Uganda

**DOI:** 10.1371/journal.pone.0247468

**Published:** 2021-02-18

**Authors:** The PLOS ONE Staff

## Notice of Republication

This article was republished on **February 5, 2021**, to correct the erroneous inclusion of figure text in the Discussion section. This error was introduced during the typesetting process. The publisher apologizes for the error. Please download this article again to view the correct version. The originally published, uncorrected article and the republished, corrected articles are provided here for reference.

## Supporting information

S1 File Originally published, uncorrected article. (PDF)

S2 File Republished, corrected article. (PDF)
