# Correction: The genetic risk for hypertension is lower among the Hungarian Roma population compared to the general population

**DOI:** 10.1371/journal.pone.0255129

**Published:** 2021-07-20

**Authors:** The PLOS ONE Staff

## Notice of republication

This article was republished on April 27 2021, to correct numerous errors in the content and placement of references and text. The following errors were addressed: values in the Table 1 References column, the number and content of two references (now [34] and [35]) in the Statistical analysis section, Fig 1 caption appearing incorrectly in the main text of the Statistical analysis section, an incorrect number and content of a reference (now [36]) in the Computations using GRS and wGRS values subsection of the Materials and methods sections, and lastly, the first and last pages missing in S1 File. These errors were introduced during the typesetting process. The publisher apologizes for the error. Please download this article again to view the correct version. The originally published, uncorrected article and the republished, corrected articles are provided here for reference.

## Supporting information

S1 File Originally published, uncorrected article. (PDF)

S2 File Republished, corrected article. (PDF)
