# Supplementary material for: Correction: The genetic risk for hypertension is lower among the Hungarian Roma population compared to the general population
Source: PLoS One. 2021 Jul 20;16(7):e0255129. doi: 10.1371/journal.pone.0255129 (PMC8291681; doi:10.1371/journal.pone.0255129)
Supplement: S1 File — (PDF) [file pone.0255129.s001.pdf]

PLOS ONE

RESEARCH ARTICLE

The genetic risk for hypertension is lower among the Hungarian Roma population compared to the general population

Beáta Soltész[1], Péter Pikó[2], János Sándor[3,4], Zsigmond Kósa[5], Róza Ádány[2,3,4], Szilvia Fiatal[3,4]*

1 Doctoral School of Health Sciences, Department of Preventive Medicine, Faculty of Public Health, University of Debrecen, Debrecen, Hungary, **2** MTA-DE Public Health Research Group of the Hungarian Academy of Sciences, Faculty of Public Health, University of Debrecen, Debrecen, Hungary, **3** Department of Preventive Medicine, Faculty of Public Health, University of Debrecen, Debrecen, Hungary, **4** WHO Collaborating Centre on Vulnerability and Health, Department of Preventive Medicine, Faculty of Public Health, University of Debrecen, Debrecen, Hungary, **5** Department of Health Visitor Methodology and Public Health, Faculty of Health, University of Debrecen, Nyíregyháza, Hungary

* fiatal.szilvia@sph.unideb.hu

Check for updates

Abstract

Estimating the prevalence of cardiovascular diseases (CVDs) and risk factors among the Roma population, the largest minority in Europe, and investigating the role of genetic or environmental/behavioral risk factors in CVD development are important issues in countries where they are significant minority. This study was designed to estimate the genetic susceptibility of the Hungarian Roma (HR) population to essential hypertension (EH) and compare it to that of the general (HG) population. Twenty EH associated SNPs (in *AGT*, *FMO3*, *MTHFR-NPPB*, *NPPA*, *NPPA-AS1*, *AGTR1*, *ADD1*, *NPR3-C5orf23*, *NOS3*, *CACNB2*, *PLCE1*, *ATP2B1*, *GNB3*, *CYP1A1-ULK3*, *UMOD* and *GNAS-EDN3*) were genotyped using DNA samples obtained from HR (N = 1176) and HG population (N = 1178) subjects assembled by cross-sectional studies. Allele frequencies and genetic risk scores (unweighted and weighted genetic risk scores (GRS and wGRS, respectively) were calculated for the study groups and compared to examine the joint effects of the SNPs. The susceptibility alleles were more frequent in the HG population, and both GRS and wGRS were found to be higher in the HG population than in the HR population (GRS: 18.98 ± 3.05 vs. 18.25 ± 2.97, p<0.001; wGRS: 1.4 [IQR: 0.93–1.89] vs. 1.52 [IQR: 0.99–2.00], p<0.01). Twenty-seven percent of subjects in the HR population were in the bottom fifth (GRS $\leq$ 16) of the risk allele count compared with 21% of those in the HG population. Thirteen percent of people in the HR group were in the top fifth (GRS $\geq$ 22) of the GRS compared with 21% of those in the HG population (p<0.001), i.e., the distribution of GRS was found to be left-shifted in the HR population compared to the HG population. The Roma population seems to be genetically less susceptible to EH than the general one. These results support preventive efforts to lower the risk of developing hypertension by encouraging a healthy lifestyle.

OPEN ACCESS

Citation: Soltész B, Pikó P, Sándor J, Kósa Z, Ádány R, Fiatal S (2020) The genetic risk for hypertension is lower among the Hungarian Roma population compared to the general population. PLoS ONE 15(6): e0234547. https://doi.org/10.1371/journal.pone.0234547

Editor: Narasimha Reddy Parine, King Saud University, SAUDI ARABIA

Received: August 6, 2019

Accepted: May 28, 2020

Published: June 17, 2020

Copyright: © 2020 Soltész et al. This is an open access article distributed under the terms of the Creative Commons Attribution License, which permits unrestricted use, distribution, and reproduction in any medium, provided the original author and source are credited.

Data Availability Statement: All relevant data are within the manuscript and its Supporting Information files.

Funding: This research was supported by the TÁMOP 4.2.2.A-11/1/KONV-2012-0031 'Social Renewal Operational Programme – IGEN-HUNGARIAN' and the GINOP-2.3.2-15- 2016-00005 project. The projects were co-financed by the European Union and the European Regional Development Fund.

Competing interests: We declare here that the above mentioned manuscript has not been published or accepted for publication elsewhere and not under editorial review for publication elsewhere; and that our institute (University of Debrecen, Faculty of Public Health, Department of Preventive Medicine) is fully aware of this submission. This does not alter our adherence to PLOS ONE policies on sharing data and materials.

Introduction

Under the leadership of the WHO, all Member States agreed in 2013 on global mechanisms to reduce the avoidable burden of noncommunicable diseases (NCDs). This plan aims to reduce the number of premature deaths from NCDs by 25% through nine voluntary global targets by 2025. Two of the global targets directly focus on preventing and controlling cardiovascular diseases (CVDs), which are the number one cause of death globally [1]. Elevated blood pressure is one of the most important independent modifiable risk factors for CVDs. Essential hypertension (EH) is the common form of hypertension and constitutes 95% of all hypertension cases [2]. Hungary still has one of the worst CVD mortality profiles when compared to other European Union countries [3]. According to recent WHO data, the early death caused by CVDs is three times more in Hungary than the European Union [4].

In our recent health examination survey [5], in which the prevalence of metabolic syndrome and its components were defined, the prevalence of hypertension was significantly lower among the Hungarian Roma (HR) population compared to the general Hungarian (HG) population (40.40% vs. 48.44%, $p<0.001$, respectively). Furthermore, a significant difference between the Hungarian Roma population and the general Hungarian population among males (43.08% vs. 53.01%, $p = 0.007$, respectively) but only a distinct trend toward significance among females (38.68% vs. 44.32%, $p = 0.063$, respectively) was found.

Roma is the largest ethnic group in Europe, with an estimated population of 10–12 million [6]. Hungary is one of the countries with the highest share of the Roma population. It was estimated by the latest national census in 2011 that approximately 3.2% of the total population of the country is Roma; notwithstanding, their estimated representation is much higher, up to 8.7% of the total population [7].

Several studies estimated the prevalence of hypertension among the Roma population compared to the majority population; however, the results were not fully consistent. Studies pointed out the lower hypertension prevalence in the Roma population compared to the general population of Romania and Croatia [8] [9] [10] [11]. Other studies, carried out in Slovakia, Spain and Italy, did not find any differences in the prevalence of hypertension among the Roma population compared to the general population [12] [13] [14] [15] [16].

EH is considered to be a polygenic and multifactorial disease influenced by environmental determinants (salt and fat intake; physical inactivity) [17]. The genetic contribution to blood pressure variation ranges from approximately 30 to 50% based on the heritability estimates of family and twin studies [18] [19]. Blood pressure (BP) is controlled by a complex network of interacting biochemical and physiological pathways (involving cardiac contractility, extracellular fluid volume homeostasis, and vascular tone through neural, renal or endocrine systems). The knowledge of these systems offered the opportunity to investigate the possible role of genes encoding proteins using a candidate gene approach [20] [21] [22]. Numerous GWASs have discovered variants in different loci within the genome that are significantly associated with blood pressure and hypertension [23]. Individual risk alleles found in GWASs and candidate gene studies explain only a very small proportion of the variation in systolic and diastolic blood pressure; consequently, the predictive value of the individual alleles for the risk of the trait are limited. Summarizing the multiple susceptible and protective alleles of single nucleotide polymorphisms (SNPs) by polygenic/genetic risk score (GRS) computation offers the opportunity to estimate the genetic load on different diseases on both the individual and population levels [24] [25] [26].

Our intention was to investigate whether disparities exist in the cumulative risk allele loads between the Hungarian Roma and the general populations, which may at least to a certain extent elucidate ethnic differences in hypertension prevalence. According to findings of this

study preventive interventions such as population-wide life-style modification and the use of genetic information for risk stratification should be specifically tailored in case of the population where genetic susceptibility to essential hypertension is found to be more defined.

Materials and methods

Study design

The subjects in this study included 1176 Hungarian Roma individuals living in segregated settlements in Northeast Hungary and 1178 individuals from the Hungarian general population. Roma subjects were derived from cross-sectional studies. Details of the sample collection can be found elsewhere [5] [27]. General individuals were collected through the country's population-based disease registry called the General Practitioners' Morbidity Sentinel Stations Programme (GPMSSP) using a stratified multistage sampling method.

Characterization of the study populations

Hungarian general population. The GPMSSP, established in 1998 to monitor the occurrence of chronic noncommunicable diseases of great public health importance, provided an adequate method for generating the Hungarian reference sample in a cost-effective way [28]. In the initial phase of the Programme, only four counties were involved (Hajdú-Bihar, Győr-Moson-Sopron, Szabolcs-Szatmár-Bereg and Zala Counties). However, later, the Programme was extended to additional regions of the country (two counties from Central Hungary, Komárom-Esztergom and Bács-Kiskun, Baranya from Southern Transdanubia, and Heves from Northern Hungary) [29].

Participants in this present study were collected at the initial four-county phase of the Programme. The source population of the study involved males and females older than 20 years of age and represented the Hungarian adult population on the basis of geographic, age and gender distributions. As a part of the initial survey (demographic and anthropometric data were collected); blood samples were also taken for routine laboratory tests and DNA isolation. On the whole, 1196 blood samples were collected for DNA preparation, out of which 1178 samples yielded good quality DNA for our present study.

Hungarian Roma population. The DNA samples of the Roma population were obtained from two cross-sectional surveys. First, a comparative health examination survey, which enrolled Roma from North-East Hungary (Hajdú-Bihar and Szabolcs-Szatmár-Bereg counties) where the majority of Roma colonies can be found, was utilized. Details of the sampling methodology and the data collection are described elsewhere [5]. As a part of the survey, medical histories and sociodemographic characteristics were recorded, and a physical examination was performed for all participants. Blood samples were taken for laboratory investigations and genotype assessments.

Second, an additional source of the Roma samples was the recently launched 'Public Health Focused Model Programme for Organising Primary Care Services Backed by a Virtual Care Service Centre', which was developed in the framework of the Swiss-Hungarian Cooperation Programme [27]. The intervention area of the Programme is found in the two most disadvantaged regions of Hungary (North Hungary and the North Great Plain). In these regions (Hajdú-Bihar, Borsod-Abaúj-Zemplén, Jász-Nagykun-Szolnok and Heves counties), one of the services that is delivered by the GPs' cluster is a health status assessment, which provided the opportunity to further increase the collection of DNA samples from the Roma population. Altogether, 1292 samples representative of the Roma population living in Northern-East Hungary by age and gender are available in our repository. The DNA pool in this study consists of 1176 randomly selected Roma individuals.

DNA isolation

DNA extraction was performed from EDTA-anticoagulated whole blood samples using a MagNA Pure LC system (Roche Diagnostics GmbH, Mannheim, Germany) with a MagNA Pure LC DNA Isolation Kit–Large Volume (Cat. No. 03310515001, Roche Diagnostics GmbH, Mannheim, Germany) according the manufacturer's instructions. Extracted DNA was eluted in 200 μl MagNA Pure LC DNA Isolation Kit–Large Volume Elution Buffer.

SNP literature search and selection criteria

Systematic literature search on the PubMed and HuGE Navigator [30] databases and on the NHGRI-EBI GWAS Catalogue [31] was conducted to identify the single-nucleotide polymorphisms (SNPs) most strongly associated with EH. Details of the systematic review search can be found in the supplementary material (Fig 1 in S1 File). The following keywords and all possible combinations were used for searching: essential hypertension, blood pressure, molecular genetics, genomics, genes, single-nucleotide polymorphism, genetic variants, gene polymorphism, common gene variants, genome-wide association study (GWAS), candidate gene study, case-control study, meta-analysis, review, association.

Studies were selected if they met the following criteria: (1) investigated the association between SNPs and EH using a statistically acceptable sample size, (2) provided information about susceptibility/protective alleles of the SNPs, (3) evaluated EH as an outcome and excluded secondary forms of hypertension or other types of monogenic hypertension, (4) defined EH as systolic blood pressure higher than 140 mmHg and/or diastolic blood pressure higher than 90 mmHg, or study individuals obtained antihypertensive medication, (5) full texts were available and written in the English language and (6) conducted in humans. Additional studies were also examined by reviewing references of the selected articles.

The adequate sample size for the study groups was computed using the online calculator OSSE (http://osse.bii.a-star.edu.sg/calculation1.php), assuming a power of 80% and an alpha level of 0.05 for a 1:1 case to control ratio. The allele frequencies for CEU (Utah Residents (CEPH) with Northern and Western Ancestry) and for GIH (Gujarati Indian from Houston, Texas) populations from the 1000 Genomes Project, Phase 3, were applied in the sample size estimation considering the fact that the Roma population arrived at the Balkans from North India and then migrated to Europe [32].

As a result of the systematic literature search, 30 SNPs were identified (Table 1, Step 1 in S1 File). During the assay design, a pool of 23 SNPs was created for genotyping by the Mutation Analysis Core Facility of the Karolinska University Hospital (Sweden). Based on data obtained in the genotyping process, 20 SNPs were chosen for allele frequency comparison and GRS computation. Effect size estimates from GWASs were available for 19 SNPs. Finally, 19 SNPs were included in the computation of the wGRS (see details of SNPs' selection process in Table 1, Steps 1–5 in S1 File).

Genotyping

The genotyping was performed on a Mass-ARRAY platform (Sequenom Inc., San Diego, CA, USA) with iPLEX Gold chemistry [47]. The validation of assays, concordance analysis and quality control analysis were conducted by the facility according to their standard protocols. The total genotyping success rate was 97.8%, resulting in genotype information for 2343 individuals (1167 general and 1176 Roma).

Table 1. List of SNPs and their loci with the effect alleles and effect size estimates included in the study.

SNP ID	Locus[a]	Chromosome	Functional consequence[b]	IUPAC code followed by nucleic acid change[c]	Effect allele	Genetic model applied in the publication	Published effect for weighting		Type of SNP	References
							OR for hypertension (95% CI; p-value)	β (mmHg) (SE; p-value)		
**rs4762**	*AGT*	1	missense variant	r = G>A	T	per allele	**1.19** (1.07–1.33; 0.002)	NA	candidate	[33] [34]
**rs5049**	*AGT*	1	2kb upstream variant	Y = C>T	A	per allele	**1.37** (1.17–1.59; 0.00006)	NA	candidate	[33]
**rs699**	*AGT*	1	missense variant	R = A>G	C	per allele	**1.20** (1.11–1.29; <0.0001)	NA	candidate	[35] [34]
**rs2266782**	*FMO3*	1	missense variant	r = G>A	A	NA	NA	NA	candidate	[36]
**rs17367504**	*MTHFR-NPPB*	1	intron variant	R = A>G	G	per allele	NA	**-0.103** (-; 2.3x10^{-10})	GWAS	[37]
**rs5068**	*NPPA*	1	3 prime UTR variant	D = A>G,T	G	per allele	**0.85** (0.79–0.92; 4x10^{-5})	NA	candidate	[38]
**rs198358**	*NPPA-AS1*	1	non coding transcript variant	y = T>C	C	per allele	**0.90** (0.85–0.95; 2x10^{-4})	NA	candidate	[38]
**rs5186**	*AGTR1*	3	3 prime UTR variant	M = A>C	C	recessive model (CC vs. AC + AA)	**7.3** (1.9–31.9; 0.0005)	NA	candidate	[39]
**rs4961**	*ADD1*	4	missense variant	E = G>A,T	T	dominant model (TT + GT vs. GG)	**1.60** (1.32–1.92; 1.09x10^{-6})	NA	candidate	[40] [41]
**rs1173771**	*NPR3-C5orf23*	5	~20kb of both *C5orf23* and *NPR3*	R = A>G	G	per allele	NA	**0.062** (-; 3.2x10^{-10})	GWAS	[37]
**rs1799983**	*NOS3*	7	missense variant	F = T>A,G	T	per allele	**1.038** (1.034–1.043; 2.63x10^{-03})	NA	GWAS	[42]
**rs2070744**	*NOS3*	7	intron variant	Y = C>T	C	per allele	**1.04** (1.038–1.041; 6.42x10^{-04})	NA	GWAS	[42]
**rs1813353**	*CACNB2(3')*	10	intron variant	y = T>C	T	per allele	NA	**0.078** (-; 6.2x10^{-10})	GWAS	[37]
**rs4373814**	*CACNB2(5')*	10	~10kb 5' of *CACNB2*	L = G>C,T	G	per allele	NA	**-0.046** (-; 8.5x10^{-8})	GWAS	[37]
**rs932764**	*PLCE1*	10	intron variant	R = A>G	G	per allele	NA	**0.055** (-; 9.4x10^{-9})	GWAS	[37]
**rs2681472**	*ATP2B1*	12	intron variant	R = A>G	A	per allele	NA	**0.15** (0.02; 1.75x10^{-11})	GWAS	[24]
**rs5443**	*GNB3*	12	synonymous variant	Y = C>T	T	per allele	**2.3** (1.7–3.3; 0.00002)	NA	candidate	[43]
**rs1378942**	*CYP1A1-ULK3*	15	intron variant	I = C>A,T	C	per allele	NA	**0.073** (-; 1.0x10^{-8})	GWAS	[37]
**rs13333226**	*UMOD*	16	intron variant	R = A>G	G	per allele	**0.87** (0.84–0.91; 3.6x10^{-11})	NA	GWAS	[44]
**rs6015450**	*GNAS-EDN3*	20	intron variant	R = A>G	G	per allele	NA	**0.11** (-; 4.2x10^{-14})	GWAS	[37]

[a]Locus data for each of the SNPs listed in the table are derived from the abovementioned references, according to gene(s) reported by the authors.

[b]Data on the functional consequences of SNPs were derived from dbSNP Build 153 database [45]. However in case of two SNPs the following data were used: in case of rs1173771 and rs4373814 SNPs the data defined by the references were used.

[c]The alleles for each of the of SNPs were extracted from the dbSNP Build 153 database [45], then the IUPAC codes of SNPs were defined manually based on Johnson, 2010 [46].

NA—not applicable

https://doi.org/10.1371/journal.pone.0234547.t001

Statistical analysis

Statistical analyses were performed using PLINK (version 1.07), STATA (version 12), Haploview (version 4.1) and MS Excel (version 2016) software. Age, gender, and body mass index (BMI) were assigned as confounding variables. A Shapiro-Wilk test for normality was performed. The nonparametric Kolmogorov-Smirnov test was used to compare the age and BMI values of the study groups. Allele frequencies were calculated on the basis of the obtained genotype distributions. The existence of Hardy-Weinberg equilibrium, the differences in allele distribution and the gender distribution were investigated with χ^2 tests. To correct for multiple testing, the Meff (effective number of independent marker loci) method was applied, which takes into account the correlation between the SNPs. For this calculation, the online software SNPSpD was used (available at http://neurogenetics.qimrberghofer.edu.au/SNPSpD) [48]. A corrected p–value of 0.0025 was obtained. Generally, a conventional p-threshold of 0.05 was applied. Linkage disequilibrium (LD) between polymorphisms was defined using the Haploview software [49]. The pairwise LD between the markers was measured and visualized in the format of r^2 (squared coefficient of correlation), with a threshold of ≥ 0.8 for the study populations (see Fig 1). In order to investigate whether the association between genetic risk and ethnicity depends on the influence of other factors (age, gender, BMI were available), multivariate linear regression analyses were conducted (see details in Computations using GRS and wGRS values section). Furthermore, using blood pressure phenotype data–which was available only in case of the Roma sample—multivariate logistic and linear regression models were developed to analyze the association of GRSs with hypertension (as binary outcome) and with systolic or diastolic blood pressure (as continuous outcome), in unadjusted and adjusted models (see details in Computations using GRS and wGRS values section).

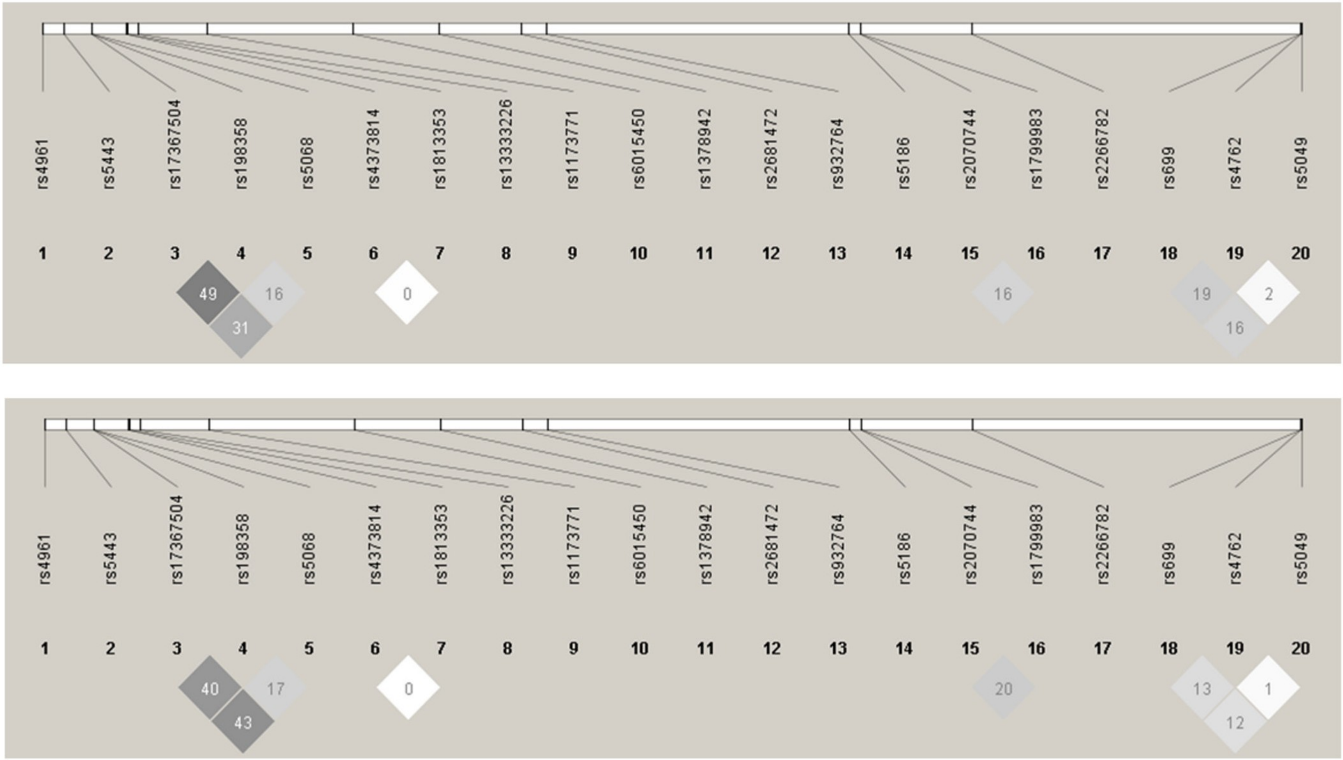

Fig 1. LD pattern of SNPs associated with hypertension for the HG (upper) and HR (lower) populations.

https://doi.org/10.1371/journal.pone.0234547.g001

Linkage analysis were conducted separately in the study populations. According to the LD map which generated by Haploview software (version 4.1), there were not observed multicollinearity between the polymorphisms, based on the LD pattern none of the pairwise LD of the studied SNPs reached the r^2 threshold of $\geq$0.8, thus it was not necessary to prune SNP from the analysis. The numbers above the LD plot show the rs numbers of SNPs. Numbers in squares are r^2 values. The colour scheme is the r^2 colour scheme (white $r^2 = 0$, shades of grey $0 < r^2 < 1$).

Computations using GRS and wGRS values

To obtain the combined effect of the selected SNPs, unweighted (GRSs) and weighted genetic risk scores (wGRSs) were calculated. For subjects whose SNP genotype data were incomplete, an expected value was imputed using the effect allele frequency observed in study populations (according to the PLINK software manual SNP scoring routine chapter, available at http://zzz.bwh.harvard.edu/plink/dist/plink-doc-1.07.pdf). Variants, their effect alleles and weights included in the GRS and wGRS computation are depicted in Table 1.

In the GRS computation, each person was assigned a score based on the number of risk alleles carried. Thus, risk allele homozygotes were coded as genotype "2", heterozygotes were coded as genotype "1", and "0" indicated the absence of the risk allele. When the effect allele was reported to be protective, the coding was "0" for effect allele homozygotes and "2" for other allele homozygotes [50]. By using these codes, a simple count score (unweighted GRS) was computed as described by Eq (1), in which G_i is the number of risk alleles for the ith SNP. This model sums all risk alleles over all loci as a summary score assuming that all alleles have the same effect.

$$GRS = \sum_{i=1}^{I} G_i \tag{1}$$

In the weighted approach (wGRS), rather than giving equal weight to each SNP, SNPs with larger effects contributed more to the score. The effect size measures were utilized from studies that published significant effect size measure results on population samples of European subjects. Risk estimates for alleles were available for 19 SNPs in 16 genes or loci (Table 1). Formerly, we showed that the effect of the vast majority of the SNPs on HDL-C levels could be replicated in the HG and Roma populations, which indicates that the effect size measurements obtained from the literature on European populations can be used for risk estimation for the Roma population [51]. In the case of the rs4762 variant, the published "pooled OR" (OR of European + Asian + Mixed populations) was used because the data for Europeans were not significant. In addition, in the same meta-analysis, the rs5049 variant was described mainly among Asians, and no evidence for heterogeneity of the effect size measure across studies was reported [33]. The computation of the wGRS is described by Eq (2). In this weighted score, weights (w_{β_i}) were derived from the risk coefficient for each allele based on beta values (or odds ratio if applicable, as the natural logarithm of the published OR for hypertension). These weights (w_{β_i}) were multiplied by 0, 1 or 2 according to the number of effect alleles carried by each person (X_i) [50] [52] [53]. In the case of two SNPs (rs4961 and rs5186), different allele coding was needed because the risk coefficients were reported for one or two copies of each effect allele as a single group (in the case of rs4961, the T allele was coded in a dominant model TT + GT vs. GG; in the case of rs5186, the C allele was coded in a recessive model CC vs. AC + CC). Consequently, the risk coefficients were multiplied by a score of 1 or 0, respectively [54].

$$wGRS = \sum_{i=1}^{I} w_{\beta\_i} X_i \tag{2}$$

The nonparametric two-sample Mann-Whitney U test and two-sample Kolmogorov-Smirnov test were used to compare the distribution of GRS and wGRS, respectively. The χ^2 test was used to compare GRSs divided into quintiles by population. To discover whether the association between genetic risk and ethnicity depends on the influence of other factors (age, gender, BMI were available), multivariate linear regression analyses were conducted in which GRSs were the dependent variable, while ethnicity, gender, age and BMI were considered as independent variables. For the wGRSs, age and BMI values were non-normally distributed in the study populations and were therefore transformed based on the two-step method described by Templeton [55].

To investigate whether the SNPs are suitable for GRS computation, regression models were applied. Using blood pressure phenotype data–only in case of the Roma sample were available—multivariate logistic and linear regression models were developed to analyze the association of GRSs with hypertension (as binary outcome) and with systolic or diastolic blood pressure (as continuous outcome), in which Model 1 was unadjusted and Model 2 was adjusted for gender, age and BMI. Hypertension status was considered as a dependent variable, while GRS, gender, age, and BMI were considered as independent variables. A binary variable for hypertension status was defined according to the consensus definition of the International Diabetes Federation (IDF) by a systolic blood pressure $\geq$130 mm Hg, a diastolic blood pressure $\geq$85 mm Hg or the use of antihypertensive medication. In the multivariate linear regression model for those subjects who were under antihypertensive treatment control, we applied an imputation method by adding a constant 10 mmHg to the measured systolic blood pressure values and 5 mmHg to diastolic values [56]. The wGRS, GRS, age, BMI, systolic and diastolic blood pressure values were non-normally distributed in the study populations and were therefore transformed [55].

Ethics statement

All procedures performed in studies involving human participants were in accordance with the ethical standards of the institutional and/or national research committee and with the 1964 Helsinki declaration and its later amendments or comparable ethical standards. All subjects gave their written informed consent for the study. This study was approved by the Ethical Committee of the Hungarian Scientific Council on Health (reference Nos. ETT-TUKEB 8907-0/2011-EKU (285/PI/11.) TUKEB 2213-5/2013/EKU (233/2013). This article does not contain any studies with animals performed by any of the authors.

Results

Characteristics of the study sample

In total, 1176 Roma and 1167 general individuals were included in the analyses. The mean age was 40.98 ± 12.84 years in the Roma population and 47.31 ± 17.02 years in the HG population. The mean age of the two study populations was different ($p<0.001$). The proportion of male individuals was lower in the Roma sample (HR: 41% vs. HG: 46%, p = 0.01). The mean body mass index (BMI) was higher in the Roma sample compared with the HG sample (26.55 ± SD 6.54 kg/m^2 vs. 26.10 ± SD 4.88 kg/m^2, $p<0.001$).

In the case of rs1799998 and rs3918226, polymorphisms deviated from HWE in the Hungarian general population ($p<0.05$) and were thus excluded from further analyses.

Comparison of allele frequencies

Allele frequency differences between the Roma and general populations are significant for 7 SNPs after multiple test correction ($p<0.0025$, Table 2). Of the 5 protective SNPs examined, only one (the rs13333226 variant of the *UMOD* gene) was significantly more frequent in the

Table 2. Comparison of protective and susceptibility allele frequencies between the Hungarian general and Roma populations.

Locus	SNPs	Allele	Hungarian general population (N = 1167)	Hungarian Roma population (N = 1176)	p-value
**Protective allele frequencies (%)**					
CACNB2(5')	rs4373814	G	52.29	55.01	0.069
MTHFR-NPPB	rs17367504	G	13.62	11.81	0.070
NPPA	rs5068	G	4.80	5.56	0.253
NPPA-AS1	rs198358	C	23.58	25.07	0.249
UMOD	**rs13333226**	G	**17.26**	11.41	**<0.001**
**Susceptibility allele frequencies (%)**					
ADD1	rs4961	T	18.39	16.47	0.092
AGT	rs4762	A	14.84	12.81	0.051
AGT	rs5049	T	13.38	11.29	0.035
AGT	rs699	G	47.88	51.01	0.038
AGTR1	**rs5186**	C	**26.22**	13.41	**<0.001**
ATP2B1	**rs2681472**	A	**83.89**	74.59	**<0.001**
CACNB2(3')	rs1813353	T	62.38	61.06	0.368
CYP1A1-ULK3	**rs1378942**	C	38.70	**49.68**	**<0.001**
FMO3	rs2266782	A	39.68	36.69	0.040
GNAS-EDN3	**rs6015450**	G	**12.86**	7.41	**<0.001**
GNB3	rs5443	T	33.02	33.90	0.537
NOS3	rs1799983	T	*31.12*	28.06	0.026
NOS3	**rs2070744**	C	**39.56**	31.16	**<0.001**
NPR3-C5orf23	rs1173771	G	58.97	55.58	0.023
PLCE1	**rs932764**	G	**46.74**	40.65	**<0.001**

SNPs in bold showed highly significantly (p<0.0025) different frequencies in the two populations after multiple test correction.

https://doi.org/10.1371/journal.pone.0234547.t002

HG population. The majority of susceptibility alleles were more prevalent in the HG population; the difference in the prevalence of five alleles (the rs5186 allele of the *AGTR1* gene, the rs2681472 allele of the *ATP2B1* gene, the rs6015450 allele at the *GNAS-EDN3* locus, the rs2070744 allele of the *NOS3* gene, and the rs932764 allele of the *PLCE1* gene) reached a high level of significance (p<0.0025). In the case of five other alleles (the rs4762 and rs5049 alleles of the *AGT* gene, the rs2266782 allele of the *FMO3* gene, the rs1799983 allele of the *NOS3* gene, and the rs1173771 allele at the *NPR3-C5orf23* locus), the significance was found to be nominal (p<0.05), and the other two SNPs (the rs4961 allele of the *ADD1* gene and the rs1813353 allele of the *CACNB2(3')* gene) did not differ significantly. Three susceptibility variants were more common among the Roma population; the difference reached a high level of significance (p<0.0025) only in the case of the rs1378942 variant at the *CYP1A1-ULK3* locus, while only nominal significance (p<0.05) was observed in the case of the rs699 variant of the *AGT* gene, and the frequency of the rs5443 variant of the *GNB3* gene did not differ significantly.

Linkage analysis

Linkage analysis was conducted separately in the study populations (Fig 1). Based on the LD pattern, none of the pairwise LDs of the studied SNPs reached the r^2 threshold of ≥0.8.

Comparison of genetic risk scores

GRS results. The GRS (based on 20 SNPs) for the HR population ranged from 9 to 26. The GRS for HG subjects ranged from 11 to 29. GRS data were normally distributed for the

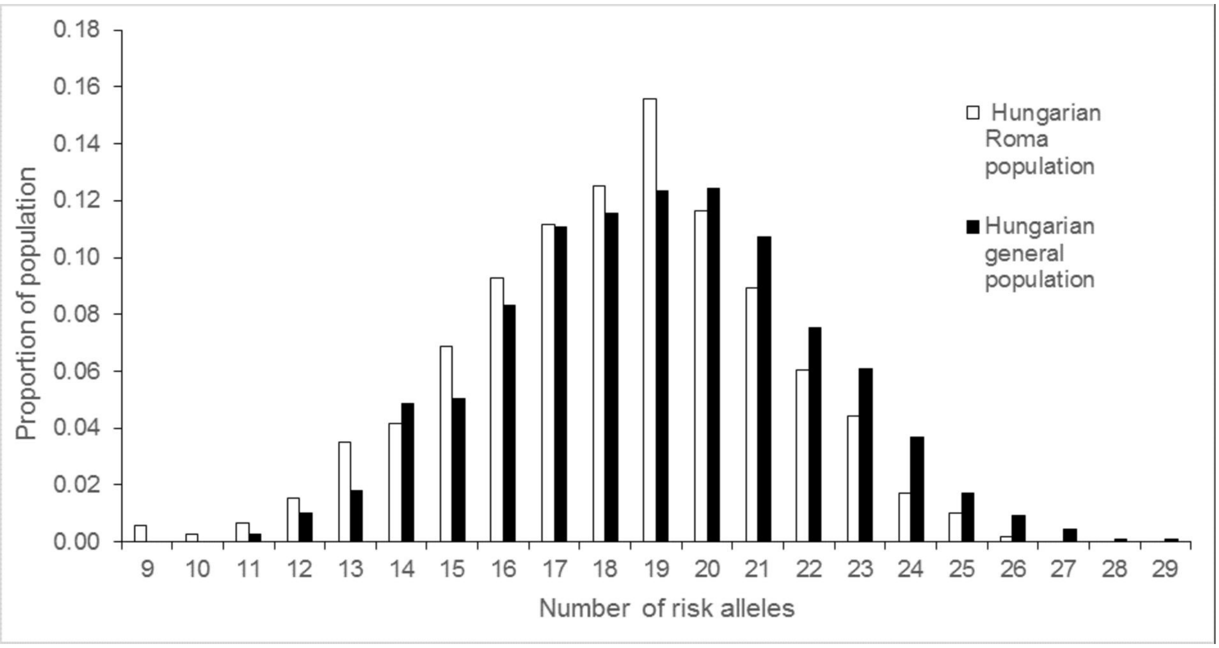

Fig 2. The distributions of GRSs in the HG (black) and HR (white) populations were significantly different (p<0.001).

https://doi.org/10.1371/journal.pone.0234547.g002

HG population, while non-normally distributed for the HR population. The mean of the gene count score was 18.25 ± SD 2.97 in the HR group and 18.98 ± SD 3.05 in the HG group. The study groups were different by GRS distribution according to the Mann-Whitney U test (p<0.001, Fig 2).

The multivariate linear regression analysis performed on the GRSs confirmed the association between genetic risk and ethnicity independently of the effect of age, gender and BMI (p<0.001, Table 3, part A).

Table 3. The multivariate linear regression analysis performed on GRSs to confirm the association between genetic risk and ethnicity.

A) dependent variable: GRS		R Square = 0.0167	
**Independent variables**	**Coefficient**	**p value**	**β**
ethnicity (General vs. Roma)	-0.742	<0.001	-0.122
gender (men vs. women)	-0.201	0.119	-0.033
age	-0.001	0.857	0.004
BMI	-0.010	0.365	-0.020
B) dependent variable: wGRS		R Square = 0.0085	
**Independent variables**	**Coefficient**	**p value**	**β**
ethnicity (General vs. Roma)	-0.125	<0.001	-0.080
gender (men vs. women)	-0.050	0.135	-0.032
age	-0.0001	0.939	-0.002
BMI	-0.004	0.150	-0.032

β: relative strength of predictors

The wGRS, age and BMI values were non-normally distributed and were transformed using a two-step approach suggested by Templeton [55]. Multivariate regression analysis using age, gender and BMI as covariates did not change the inference neither for the GRS nor for wGRS.

https://doi.org/10.1371/journal.pone.0234547.t003

Table 4. Association between GRSs and hypertension risk in the Hungarian Roma population.

A) dependent variable: Hypertension status				
**model No.**	**independent variables**	**OR**	**95% CI**	**p value**
Model 1[a]	**GRS**	1.05	1.01–1.09	0.027
Model 2[b]	**GRS**	1.07	1.02–1.12	0.008
	**gender** (men vs. women)	0.91	0.68–1.22	0.530
	**age**	1.09	1.08–1.11	<0.001
	**BMI**	1.14	1.11–1.17	<0.001
**B) dependent variable: Hypertension status**				
**model No.**	**independent variables**	**OR**	**95% CI**	**p value**
Model 1[a]	**wGRS**	1.06	0.91–1.25	0.457
Model 2[b]	**wGRS**	1.12	0.92–1.37	0.248
	**gender** (men vs. women)	0.90	0.67–1.21	0.504
	**age**	1.09	1.08–1.11	<0.001
	**BMI**	1.14	1.11–1.16	<0.001

[a]Model 1 is unadjusted

[b]Model 2 is adjusted for gender, age and BMI

https://doi.org/10.1371/journal.pone.0234547.t004

The multivariate logistic regression analysis showed that carrying 1 additional risk allele in the Roma study subjects was associated with a 7% increase in the odds of hypertension, independently of the effect of age, gender and BMI (OR = 1.07; 95% CI: 1.02–1.12; p = 0.008, Table 4, part A).

The GRSs were significantly associated with systolic (β = 0.401 mmHg, 95% CI 0.052–0.750, p = 0.024) but not with diastolic blood pressure (β = 0.149 mmHg, 95% CI—0.045–0.344, p = 0.132) in the multivariate linear regression analyses (Table 5, part A)

Twenty-seven percent of subjects in the HR population were in the bottom fifth (GRS ≤ 16) of the gene count score compared with 21% of those in the HG population. Thirteen percent of people in the HR group were in the top fifth (GRS ≥ 22) of the GRSs compared with 21% of those in the HG population (p<0.001), i.e., the distribution of GRSs was found to be shifted to the left in the HR population compared to the HG population.

wGRS results. The applicable effect size estimate for the rs2266782 allele in the *FMO3* gene was not publicly available; therefore, 19 SNPs were used for the weighted genetic risk score computation (Table 1). The HR study group had a lower weighted genetic risk than the HG group, as the median wGRS in the HR group was 1.40 (IQR: 0.93–1.89), while for the HG individuals, the median wGRS was 1.52 (IQR: 0.99–2.00). The lower HR median suggests that the HR population has a lower weighted genetic risk score on average (p<0.01). The boxplot of the wGRSs estimated from the study populations (HG and HR) is shown in Fig 3. wGRS data were non-normally distributed with skewness of 0.56 (SE = 0.07) and kurtosis of 0.59 (SE = 0.14) in the HR population and with skewness of 0.79 (SE = 0.07) and kurtosis of 0.91 (SE = 0.14) in the HG population.

The multivariate linear regression model was able to confirm the association between genetic risk and ethnicity independently of the effect of age, gender and BMI (p<0.001, Table 3, part B).

The multivariate logistic regression analysis was not able to confirm the association of wGRS with hypertension risk in the Hungarian Roma population (OR = 1.12; 95% CI 0.92–1.37; p = 0.248, Table 4, part B).

The weighted genetic risk score was significantly associated with both systolic (β = 1.906, 95% CI 0.572–3.240, p = 0.005) and diastolic blood pressure (β = 1.035, 95% CI: 0.293–1.778, p = 0.006) in the multivariate linear regression analyses (Table 5, part B).

Table 5. The association of GRSs were with systolic and diastolic blood pressure.

model No.	independent variables	coefficient	95% CI	p value	β
**A) dependent variable: Systolic blood pressure**					
Model 1[a]	**GRS**	0.432	-0.8631	0.05	-
Model 2[b]	**GRS**	0.401	0.052–0.750	0.024	0.055
	**age**	0.683	0.600–0.765	<0.001	0.403
	**gender** (men v. women)	-5.415	-4.175	<0.001	-0.124
	**BMI**	1.143	0.981–1.305	<0.001	0.346
**dependent variable: Diastolic blood pressure**					
Model 1[a]	**GRS**	0.151	-0.449	0.186	-
Model 2[b]	**GRS**	0.149	-0.389	0.132	0.039
	**age**	0.291	0.245–0.338	<0.001	0.331
	**gender** (men vs. women)	-1.966	-2.325	0.001	-0.086
	**BMI**	0.551	0.461–0.641	<0.001	0.321
**B) dependent variable: Systolic blood pressure**					
Model 1[a]	**wGRS**	1.794	0.137–3.450	0.034	-
Model 2[b]	**wGRS**	1.906	0.572–3.240	0.005	0.068
	**age**	0.682	0.600–0.765	<0.001	0.403
	**gender** (men vs. women)	-5.356	-4.171	<0.001	-0.122
	**BMI**	1.147	0.985–1.308	<0.001	0.347
**dependent variable: Diastolic blood pressure**					
Model 1[a]	**wGRS**	0.983	0.123–1.843	0.025	-
Model 2[b]	**wGRS**	1.035	0.293–1.778	0.006	0.071
	**age**	0.291	0.245–0.337	<0.001	0.331
	**gender** (men vs. women)	-1.926	-2.321	0.001	-0.085
	**BMI**	0.553	0.463–0.643	<0.001	0.322

β: relative strength of predictors

[a]Model 1 is unadjusted

[b]Model 2 is adjusted for age, gender and BMI

The GRS, wGRS, age, BMI, systolic and diastolic blood pressure values were non-normally distributed and were transformed using a two-step approach suggested by Templeton [55].

https://doi.org/10.1371/journal.pone.0234547.t005

A total 21.51% of Roma subjects were in the bottom quintile (wGRS ≤-0.54) of the wGRSs compared with 18.51% of those in the general population. In the Roma group, 18.37% of the individuals were in the highest quintile (wGRS≥4.92) of the wGRSs compared with 21.59% of those in the general population (p = 0.029) (Table 2 in S1 File).

Discussion

EH is a well-established risk factor for adverse cardiovascular outcomes. Estimating the prevalence of cardiovascular risk factors among the Roma population and investigating the role of genetic or environmental/behavioral/cultural risk factors in the development of CVDs is an important issue in countries where they are significant minorities. Our study is the first to examine the genetic susceptibility of the Roma population to EH by comparing allele frequencies and combining the effect of multiple hypertension-associated alleles into GRSs. In addition, the number of Roma subjects surveyed is relatively high.

Analysis of the biological, environmental, social and physiological domains related to ethnicity is an essential component of the integrative research process that aims to prevent the development of disease, including those that differ in prevalence among ethnic groups. Some

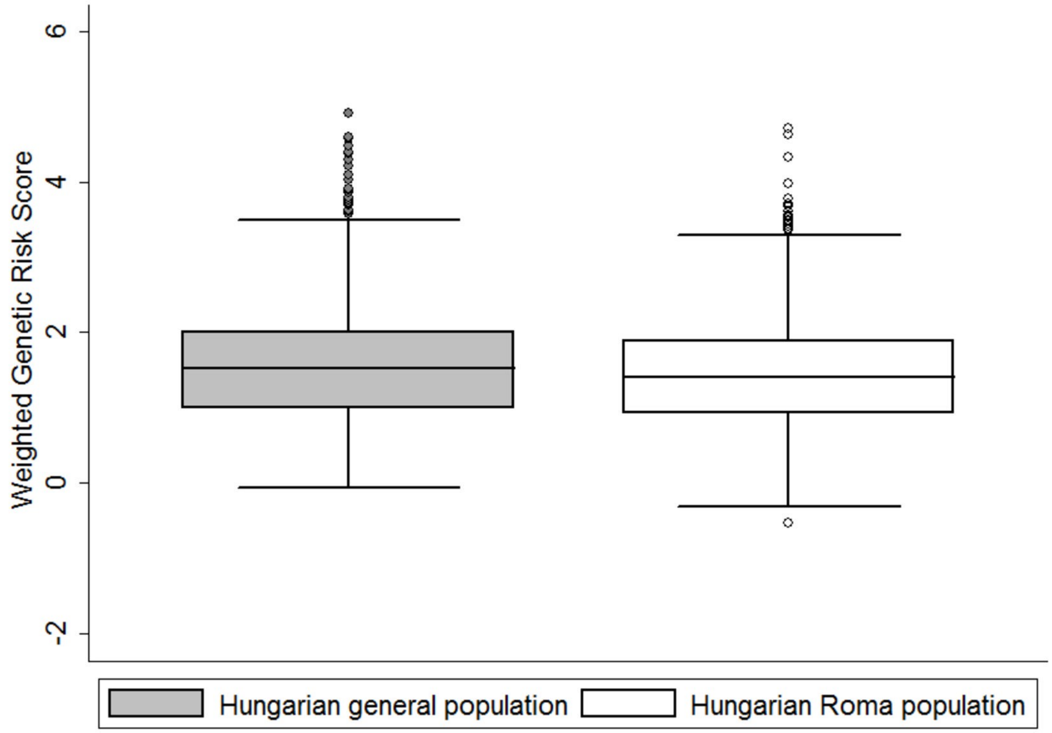

Fig 3. Distributions of wGRSs in the HG (grey) and HR populations (white) were significantly different (p<0.01).

https://doi.org/10.1371/journal.pone.0234547.g003

studies have already reported that the cardiovascular risk load in the Roma population differs from the majority population of the country where they live [8] [11]. Only a few studies have investigated the possible role of genetic factors associated with cardiovascular traits [57] [58], and the genetic load of the Roma population related to hypertension has not been investigated until now.

In summary, the susceptibility alleles showing significant differences in their frequency between the two populations were more prevalent in the HG population. In addition to the simple unweighted scores, effect size estimates from GWASs were utilized to model and compare the genetic risk related to hypertension in the two study populations. It was shown that the average risk scores (both GRSs and wGRSs) were significantly lower among the Roma population compared to the HG population even after adjusting for the effects of possible confounders. The multivariate linear regression analysis performed on the GRSs was able to confirm the association between genetic risk and ethnicity independently of the effect of age, gender and BMI. In conclusion, decreased genetic susceptibility to hypertension in the Roma was observed compared to the HG population.

Despite its uniqueness, there are obvious limitations of this study. First, the list of data collected for the representative sample of the general HG was limited because data on blood pressure were not included in the analyses. Consequently, it was not possible to investigate the strengths of the association of different blood pressure phenotypes (systolic and diastolic blood pressure) with the GRSs by regression models. Another disadvantage is that the Roma study population was not representative of the overall Roma population of Hungary because those Roma who have assimilated with the general population were excluded by the sampling

method. However, because many people unwilling to self-report their ethnicity as Roma this pressure would be very difficult to manage. Third, unweighted GRS models have limitations. It assumes that alleles have a separate additive effect, and neither the effect sizes nor their possible interactions are noted. Furthermore, effect of epigenetic factors, or other structural/rare variants, and interactions (gene-environmental and gene-gene) were not taken into consideration in this study; however, it is accepted that these factors can alter genetic risk. It is important to point out that studies are needed to ascertain the impact not only of the genetic components but also of the interactions between inheritable an additionally environmental factors in the interpretation of ethnic disparities.

For the wGRS effect, size measures obtained from the population of European ancestry were applied. According to the catalogue of published genome-wide association studies (NHGRI-EBI), the GWASs related to EH have been conducted mostly on populations of European and African American descent. Additionally, effect size estimates relevant to Roma population from candidate gene studies are unavailable. Although the question as to what extent the effect size measures estimated among Caucasians are relevant for the Roma population in genetic risk assessment for essential hypertension, it is important to refer to our recent study on HDL-C levels associated genetic loci. We demonstrated that effect size estimates obtained from genome wide association studies can be utilized for risk estimation not only for the general population but also in the case of the Roma.

Applying multiple markers in combination instead of using single SNPs with small effect size can be more advantageous if we would like to translate results from genomic studies to population health research. In the genomic era, identifying people or populations who are at high risk of developing common CVDs may augment the advantage of prevention programmes by decreasing the risk of the development of CVDs. It is important to highlight that more research is needed because, so far, only limited evidence is available on the application of genetic/genomic results in public health practice [59]. Our present finding suggests that prevention of EH in the Roma population should focus on harmful environmental or behavioral factors rather than their genetic propensity.

Supporting information

S1 File.
(PDF)

Author Contributions

Conceptualization: Zsigmond Kósa, Róza Ádány, Szilvia Fiatal.

Formal analysis: Beáta Soltész, Péter Pikó.

Investigation: Zsigmond Kósa.

Methodology: Péter Pikó, János Sándor, Zsigmond Kósa.

Supervision: Róza Ádány, Szilvia Fiatal.

Validation: Szilvia Fiatal.

Visualization: Beáta Soltész, Péter Pikó, Róza Ádány.

Writing – original draft: Beáta Soltész, Szilvia Fiatal.

Writing – review & editing: Róza Ádány, Szilvia Fiatal.

References

1. Organization WH. Global action plan for the prevention and control of noncommunicable diseases 2013–2020. available at: https://www.who.int/nmh/publications/ncd-action-plan/en/. 2013.
2. Carretero OA, Oparil S. Essential hypertension. Part I: definition and etiology. Circulation. 2000; 101 (3):329–35. https://doi.org/10.1161/01.cir.101.3.329 PMID: 10645931
3. Helis E, Augustincic L, Steiner S, Chen L, Turton P, Fodor JG. Time trends in cardiovascular and all-cause mortality in the 'old' and 'new' European Union countries. Eur J Cardiovasc Prev Rehabil. 2011; 18(3):347–59. https://doi.org/10.1177/1741826710389361 PMID: 21450659
4. WHO Regional Office for Europe Ehfad, Copenhagen, Denmark. 2019. July.
5. Kosa Z, Moravcsik-Kornyicki A, Dioszegi J, Roberts B, Szabo Z, Sandor J, et al. Prevalence of metabolic syndrome among Roma: a comparative health examination survey in Hungary. Eur J Public Health. 2015; 25(2):299–304. https://doi.org/10.1093/eurpub/cku157 PMID: 25231955
6. Communication from the Commission to the European Parliament, the Council, the European Economic and Social Committee, and the Committee of the Regions: An EU Framework for National Roma Integration Strategies up to 2020. Brussels: European Commission; 2011. available at: http://eur-lex.europa.eu/legal-content/EN/TXT/PDF/?uri=CELEX:52011DC0173&from=HR Accessed 15 July 2016.
7. Pénzes J, Tátrai P, Pásztor IZ. A roma népesség területi megoszlásának változása Magyarországon az elmúlt évtizedekben. Területi Statisztika. 2018; 58(1):3–26.
8. Enache G, Rusu E, Ilinca A, Rusu F, Costache A, Radulian G. Prevalence of Obesity and Newly Diagnosed Diabetes in the Roma Population from a County in the South Part of Romania (Călăraşi County) -Preliminary Results. Rom J Diabetes Nutr Metab Dis. 2016; 23(1):27–36.
9. Dorobantu M, Bartos D, Badila E, Oprea G, Ghiorghe S, Lungu R, et al. PREVALENCE OF ARTERIAL HYPERTENSION AND OTHER CARDIOVASCULAR RISK FACTORS IN AN ETHNIC GROUP VERSUS ROMANIAN ADULT POPULATION—ARE THEY DIFFERENT?: PP.16.28[Abstract]. Journal of Hypertension 2011; 29(Supplement A):e298.
10. Zeljko H, Skaric-Juric T, Narancic NS, Salihovic MP, Klaric IM, Barbalic M, et al. Traditional CVD risk factors and socio-economic deprivation in Roma minority population of Croatia. Coll Antropol. 2008; 32 (3):667–76. PMID: 18982735
11. Zeljko HM, Skaric-Juric T, Narancic NS, Baresic A, Tomas Z, Petranovic MZ, et al. Age trends in prevalence of cardiovascular risk factors in Roma minority population of Croatia. Econ Hum Biol. 2013; 11 (3):326–36. https://doi.org/10.1016/j.ehb.2012.02.007 PMID: 22633739
12. Vozarova de Courten B, de Courten M, Hanson RL, Zahorakova A, Egyenes HP, Tataranni PA, et al. Higher prevalence of type 2 diabetes, metabolic syndrome and cardiovascular diseases in gypsies than in non-gypsies in Slovakia. Diabetes Res Clin Pract. 2003; 62(2):95–103. https://doi.org/10.1016/s0168-8227(03)00162-1 PMID: 14581146
13. Babinska I, Veselska ZD, Bobakova D, Pella D, Panico S, Reijneveld SA, et al. Is the cardiovascular risk profile of people living in Roma settlements worse in comparison with the majority population in Slovakia? Int J Public Health. 2013; 58(3):417–25. https://doi.org/10.1007/s00038-013-0463-4 PMID: 23564005
14. Krajcovicova-Kudlackova M, Blazicek P, Spustova V, Valachovicova M, Ginter E. Cardiovascular risk factors in young Gypsy population. Bratisl Lek Listy. 2004; 105(7–8):256–9. PMID: 15543846
15. Carrasco-Garrido P, Lopez de Andres A, Hernandez Barrera V, Jimenez-Trujillo I, Jimenez-Garcia R. Health status of Roma women in Spain. Eur J Public Health. 2011; 21(6):793–8. https://doi.org/10.1093/eurpub/ckq153 PMID: 20943990
16. Gualdi-Russo E, Zironi A, Dallari GV, Toselli S. Migration and health in Italy: a multiethnic adult sample. J Travel Med. 2009; 16(2):88–95. https://doi.org/10.1111/j.1708-8305.2008.00280.x PMID: 19335807
17. Dickinson HO, Mason JM, Nicolson DJ, Campbell F, Beyer FR, Cook JV, et al. Lifestyle interventions to reduce raised blood pressure: a systematic review of randomized controlled trials. J Hypertens. 2006; 24(2):215–33. https://doi.org/10.1097/01.hjh.0000199800.72563.26 PMID: 16508562
18. Snieder H, Hayward CS, Perks U, Kelly RP, Kelly PJ, Spector TD. Heritability of central systolic pressure augmentation: a twin study. Hypertension. 2000; 35(2):574–9. https://doi.org/10.1161/01.hyp.35.2.574 PMID: 10679500
19. Miall WE, Oldham PD. The hereditary factor in arterial blood-pressure. Br Med J. 1963; 1(5323):75–80. https://doi.org/10.1136/bmj.1.5323.75 PMID: 13935402
20. Timberlake DS, O'Connor DT, Parmer RJ. Molecular genetics of essential hypertension: recent results and emerging strategies. Curr Opin Nephrol Hypertens. 2001; 10(1):71–9. https://doi.org/10.1097/00041552-200101000-00012 PMID: 11195056

21. Tanira MO, Al Balushi KA. Genetic variations related to hypertension: a review. J Hum Hypertens. 2005; 19(1):7–19. https://doi.org/10.1038/sj.jhh.1001780 PMID: 15361889

22. Singh M, Singh AK, Pandey P, Chandra S, Singh KA, Gambhir IS. Molecular genetics of essential hypertension. Clin Exp Hypertens. 2016; 38(3):268–77. https://doi.org/10.3109/10641963.2015.1116543 PMID: 27028574

23. Zheng J, Rao DC, Shi G. An update on genome-wide association studies of hypertension. Appl Inform. 2015; 2:10.

24. Levy D, Ehret GB, Rice K, Verwoert GC, Launer LJ, Dehghan A, et al. Genome-wide association study of blood pressure and hypertension. Nat Genet. 2009; 41(6):677–87.

25. Wray NR, Goddard ME, Visscher PM. Prediction of individual genetic risk to disease from genome-wide association studies. Genome Res. 2007; 17(10):1520–8. https://doi.org/10.1101/gr.6665407 PMID: 17785532

26. Humphries SE, Yiannakouris N, Talmud PJ. Cardiovascular disease risk prediction using genetic information (gene scores): is it really informative? Curr Opin Lipidol. 2008; 19(2):128–32. https://doi.org/10.1097/MOL.0b013e3282f5283e PMID: 18388692

27. Adany R, Kosa K, Sandor J, Papp M, Furjes G. General practitioners' cluster: a model to reorient primary health care to public health services. Eur J Public Health. 2013; 23(4):529–30. https://doi.org/10.1093/eurpub/ckt095 PMID: 23882116

28. Szeles G, Voko Z, Jenei T, Kardos L, Pocsai Z, Bajtay A, et al. A preliminary evaluation of a health monitoring programme in Hungary. Eur J Public Health. 2005; 15(1):26–32. https://doi.org/10.1093/eurpub/cki107 PMID: 15788800

29. Szigethy E, Szeles G, Horvath A, Hidvegi T, Jermendy G, Paragh G, et al. Epidemiology of the metabolic syndrome in Hungary. Public Health. 2012; 126(2):143–9. https://doi.org/10.1016/j.puhe.2011.11.003 PMID: 22226972

30. Yu W, Gwinn M, Clyne M, Yesupriya A, Khoury MJ. A navigator for human genome epidemiology. Nat Genet. 2008; 40(2):124–5. https://doi.org/10.1038/ng0208-124 PMID: 18227866

31. Welter D, MacArthur J, Morales J, Burdett T, Hall P, Junkins H, et al. The NHGRI GWAS Catalog, a curated resource of SNP-trait associations. Nucleic Acids Res. 2014; 42(Database issue):D1001–6. https://doi.org/10.1093/nar/gkt1229 PMID: 24316577

32. Gresham D, Morar B, Underhill PA, Passarino G, Lin AA, Wise C, et al. Origins and divergence of the Roma (gypsies). Am J Hum Genet. 2001; 69(6):1314–31. https://doi.org/10.1086/324681 PMID: 11704928

33. Gabriel S, Ziaugra L, Tabbaa D. SNP genotyping using the Sequenom MassARRAY iPLEX platform. Curr Protoc Hum Genet. 2009;Chapter 2:Unit 2 12.

34. Nyholt DR. A simple correction for multiple testing for single-nucleotide polymorphisms in linkage disequilibrium with each other. Am J Hum Genet. 2004; 74(4):765–9. https://doi.org/10.1086/383251 PMID: 14997420

35. Barrett JC, Fry B, Maller J, Daly MJ. Haploview: analysis and visualization of LD and haplotype maps. Bioinformatics. 2005; 21(2):263–5. https://doi.org/10.1093/bioinformatics/bth457 PMID: 15297300

36. Bushueva O, Solodilova M, Churnosov M, Ivanov V, Polonikov A. The Flavin-Containing Monooxygenase 3 Gene and Essential Hypertension: The Joint Effect of Polymorphism E158K and Cigarette Smoking on Disease Susceptibility. Int J Hypertens. 2014; 2014:712169. https://doi.org/10.1155/2014/712169 PMID: 25243081

37. International Consortium for Blood Pressure Genome-Wide Association S, Ehret GB, Munroe PB, Rice KM, Bochud M, Johnson AD, et al. Genetic variants in novel pathways influence blood pressure and cardiovascular disease risk. Nature. 2011; 478(7367):103–9. https://doi.org/10.1038/nature10405 PMID: 21909115

38. Newton-Cheh C, Larson MG, Vasan RS, Levy D, Bloch KD, Surti A, et al. Association of common variants in NPPA and NPPB with circulating natriuretic peptides and blood pressure. Nat Genet. 2009; 41(3):348–53. https://doi.org/10.1038/ng.328 PMID: 19219041

39. Wang WY, Zee RY, Morris BJ. Association of angiotensin II type 1 receptor gene polymorphism with essential hypertension. Clin Genet. 1997; 51(1):31–4. https://doi.org/10.1111/j.1399-0004.1997.tb02410.x PMID: 9084931

40. Cusi D, Barlassina C, Azzani T, Casari G, Citterio L, Devoto M, et al. Polymorphisms of alpha-adducin and salt sensitivity in patients with essential hypertension. Lancet. 1997; 349(9062):1353–7. https://doi.org/10.1016/S0140-6736(97)01029-5 PMID: 9149697

41. Liu K, Liu J, Huang Y, Liu Y, Lou Y, Wang Z, et al. Alpha-adducin Gly460Trp polymorphism and hypertension risk: a meta-analysis of 22 studies including 14303 cases and 15961 controls. PLoS One. 2010; 5(9).

42. Salvi E, Kutalik Z, Glorioso N, Benaglio P, Frau F, Kuznetsova T, et al. Genomewide association study using a high-density single nucleotide polymorphism array and case-control design identifies a novel essential hypertension susceptibility locus in the promoter region of endothelial NO synthase. Hypertension. 2012; 59(2):248–55. https://doi.org/10.1161/HYPERTENSIONAHA.111.181990 PMID: 22184326

43. Benjafield AV, Jeyasingam CL, Nyholt DR, Griffiths LR, Morris BJ. G-protein beta3 subunit gene (GNB3) variant in causation of essential hypertension. Hypertension. 1998; 32(6):1094–7. https://doi.org/10.1161/01.hyp.32.6.1094 PMID: 9856980

44. Padmanabhan S, Melander O, Johnson T, Di Blasio AM, Lee WK, Gentilini D, et al. Genome-wide association study of blood pressure extremes identifies variant near UMOD associated with hypertension. PLoS Genet. 2010; 6(10):e1001177. https://doi.org/10.1371/journal.pgen.1001177 PMID: 21082022

45. Sherry ST, Ward MH, Kholodov M, Baker J, Phan L, Smigielski EM, et al. dbSNP: the NCBI database of genetic variation. Nucleic Acids Res. 2001; 29(1):308–11. https://doi.org/10.1093/nar/29.1.308 PMID: 11125122

46. Johnson AD. An extended IUPAC nomenclature code for polymorphic nucleic acids. Bioinformatics. 2010; 26(10):1386–9. https://doi.org/10.1093/bioinformatics/btq098 PMID: 20202974

47. Pereira TV, Nunes AC, Rudnicki M, Yamada Y, Pereira AC, Krieger JE. Meta-analysis of the association of 4 angiotensinogen polymorphisms with essential hypertension: a role beyond M235T? Hypertension. 2008; 51(3):778–83. https://doi.org/10.1161/HYPERTENSIONAHA.107.100370 PMID: 18227406

48. Jeunemaitre X, Soubrier F, Kotelevtsev YV, Lifton RP, Williams CS, Charru A, et al. Molecular basis of human hypertension: role of angiotensinogen. Cell. 1992; 71(1):169–80. https://doi.org/10.1016/0092-8674(92)90275-h PMID: 1394429

49. Kunz R, Kreutz R, Beige J, Distler A, Sharma AM. Association between the angiotensinogen 235T-variant and essential hypertension in whites: a systematic review and methodological appraisal. Hypertension. 1997; 30(6):1331–7. https://doi.org/10.1161/01.hyp.30.6.1331 PMID: 9403549

50. Talmud PJ, Hingorani AD, Cooper JA, Marmot MG, Brunner EJ, Kumari M, et al. Utility of genetic and non-genetic risk factors in prediction of type 2 diabetes: Whitehall II prospective cohort study. BMJ. 2010; 340:b4838. https://doi.org/10.1136/bmj.b4838 PMID: 20075150

51. Piko P, Fiatal S, Kosa Z, Sandor J, Adany R. Generalizability and applicability of results obtained from populations of European descent regarding the effect direction and size of HDL-C level-associated genetic variants to the Hungarian general and Roma populations. Gene. 2019; 686:187–93. https://doi.org/10.1016/j.gene.2018.11.067 PMID: 30468910

52. Sebastiani P, Solovieff N, Sun JX. Naive Bayesian Classifier and Genetic Risk Score for Genetic Risk Prediction of a Categorical Trait: Not so Different after all! Front Genet. 2012; 3:26. https://doi.org/10.3389/fgene.2012.00026 PMID: 22393331

53. Johnson T. Efficient calculation for Multi-SNP Genetic Risk Score, in American Society of Human Genetics Annual Meeting. 2012. San Francisco, November 6–10.

54. Che R, Motsinger-Reif AA. Evaluation of genetic risk score models in the presence of interaction and linkage disequilibrium. Front Genet. 2013; 4:138. https://doi.org/10.3389/fgene.2013.00138 PMID: 23888168

55. Templeton GF. A Two-Step Approach for Transforming Continuous Variables to Normal: Implications and Recommendations for IS Research. Communications of the Association for Information Systems. 2011; 28, Article 4.

56. Cui JS, Hopper JL, Harrap SB. Antihypertensive treatments obscure familial contributions to blood pressure variation. Hypertension. 2003; 41(2):207–10. https://doi.org/10.1161/01.hyp.0000044938.94050.e3 PMID: 12574083

57. Fiatal S, Piko P, Kosa Z, Sandor J, Adany R. Genetic profiling revealed an increased risk of venous thrombosis in the Hungarian Roma population. Thromb Res. 2019; 179:37–44. https://doi.org/10.1016/j.thromres.2019.04.031 PMID: 31078119

58. Piko P, Fiatal S, Kosa Z, Sandor J, Adany R. Genetic factors exist behind the high prevalence of reduced high-density lipoprotein cholesterol levels in the Roma population. Atherosclerosis. 2017; 263:119–26. https://doi.org/10.1016/j.atherosclerosis.2017.05.028 PMID: 28624686

59. Fiatal S, Adany R. Application of Single-Nucleotide Polymorphism-Related Risk Estimates in Identification of Increased Genetic Susceptibility to Cardiovascular Diseases: A Literature Review. Front Public Health. 2017; 5:358. https://doi.org/10.3389/fpubh.2017.00358 PMID: 29445720
